# Low-Dimensional Vanadium-Based High-Voltage Cathode Materials for Promising Rechargeable Alkali-Ion Batteries

**DOI:** 10.3390/ma17030587

**Published:** 2024-01-25

**Authors:** Wei Ni

**Affiliations:** State Key Laboratory of Vanadium and Titanium Resources Comprehensive Utilization, ANSTEEL Research Institute of Vanadium & Titanium (Iron & Steel), Chengdu 610031, China; niwei@iccas.ac.cn or max.ni@hotmail.com

**Keywords:** vanadium-based materials, low-dimensional, nanomaterials, high-voltage cathodes, alkali-ion batteries, alkali-metal-ion batteries, energy storage and conversion, vanadium oxides, vanadium phosphates, vanadium fluorophosphates

## Abstract

Owing to their rich structural chemistry and unique electrochemical properties, vanadium-based materials, especially the low-dimensional ones, are showing promising applications in energy storage and conversion. In this invited review, low-dimensional vanadium-based materials (including 0D, 1D, and 2D nanostructures of vanadium-containing oxides, polyanions, and mixed-polyanions) and their emerging applications in advanced alkali-metal-ion batteries (e.g., Li-ion, Na-ion, and K-ion batteries) are systematically summarized. Future development trends, challenges, solutions, and perspectives are discussed and proposed. Mechanisms and new insights are also given for the development of advanced vanadium-based materials in high-performance energy storage and conversion.

## 1. Introduction

Vanadium-based materials have been considered one of the most promising cathode candidates for next-generation secondary batteries, especially sodium-ion batteries (SIBs) and potassium-ion batteries (PIBs), due to the merits of rich structural chemistry, high voltage output (up to over 4.0 V), cost-effectiveness, and sustainability [1,2,3]. The multivalent state of vanadium and the open framework or layered structure of vanadium-based materials are significantly beneficial to their superior performances, not only on the aspects of (rate) capacities but also the ultralong cycling stability of high-voltage alkali-ion batteries (AIBs, A = Li, Na, and K; namely, LIBs, SIBs, and PIBs) [3,4]. Polyanionic materials, e.g., phosphates, fluorophosphates, and pyrophosphates, mixed phosphates, are structurally diverse compounds, and the vanadium-containing phosphates and fluorophosphates such as Na_3_V_3_(PO_4_)_3_ and Na_3_V_2_(PO_4_)_2_F_3_ are among the most intensively studied high-voltage cathode candidate materials for promising commercial applications [5,6,7]. Low-dimensional (e.g., 0D, 1D, and 2D) materials possess unique advantages, including a high surface-area-to-volume ratio, shortened ion diffusion lengths, a larger electrolyte–electrode contact area, reduced charge–discharge time, enhanced buffering against stress and volume change, and efficient electron transport along the longitudinal direction (especially for 1D materials) [8,9].

Considering the superior performances and special merits of low-dimensional vanadium-based materials for high-voltage and high-performance cathodes of next-generation alkali-metal or alkali-metal-ion batteries, we herein timely reviewed and summarized the recent enormous and exciting results and hope to give a comprehensive overview and give insights into the emerging high-voltage metal-ion batteries for advanced energy storage and conversion.

## 2. Compositions, Structures, and Methods

### 2.1. Chemical Composition and Crystal Structure

Vanadium-based materials are generally layered or tunnel-type materials and thus are typically intercalation hosts for alkali ions, characterized by their intercalation-deintercalation (or insertion-extraction) mechanism and electrochemically long plateaus as cathode materials for alkali-ion batteries [10,11]. In operando X-ray diffraction (also called in situ XRD) is an effective way to analyze the underlying crystal structure during cell cycling [12,13], thus facilely relating the electrochemical processes with crystal structure and phase transformation for revealing the (de)intercalation mechanisms, degradation mechanisms, and optimizing the performances [14,15].

**Figure 1:**
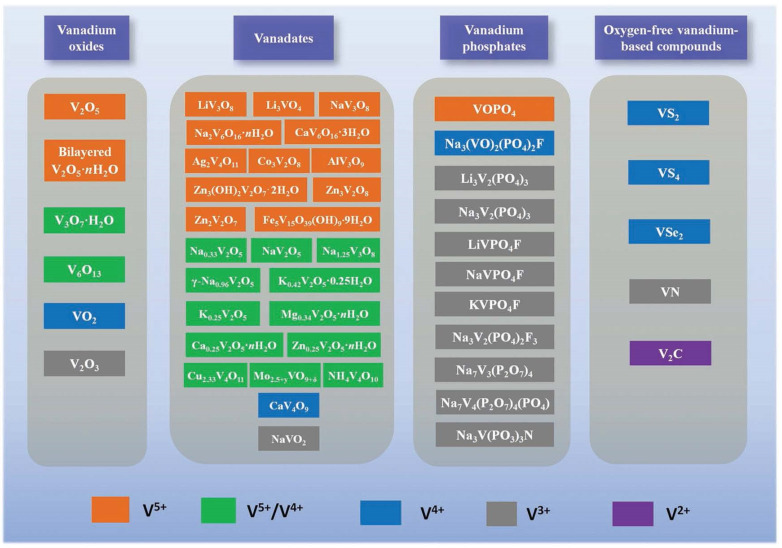
A classification of vanadium-based electrode materials for electrochemical energy storage. Adapted with permission from Ref. [3]. Copyright 2020 WILEY-VCH Verlag GmbH and Co. KGaA, Weinheim.

Vanadium oxides, vanadates, and vanadium phosphates are among the high-voltage cathode materials and have received intensive attention for electrochemical energy storage and conversion, especially for secondary batteries, in the last two decades (Figure 1) [3]. And these vanadium-based materials are evaluated in detail, as follows:

Oxygen-free vanadium-based chalcogenides, nitrides, and carbides (e.g., MXenes) are novel 2D materials with high-capacity, high-conductivity, and/or high-stability advantages; however, they are usually comparatively inferior in voltage output or generally investigated as electrode materials for supercapacitors [16,17,18]. Polyanion-based phosphates/fluorophosphates [19,20] and layered transition-metal compounds (e.g., different types of O2, O3, P2, and P3, referring to edge- and face-sharing structures [21,22,23,24]) are characterized by their sodium super ionic conductor (NASICON)-type structures for promising high-voltage cathode materials. And for vanadium-based phosphates/fluorophosphates, (transition-)metal doping/substitution (e.g., Mn, Fe, Ni, Cr, Ce, Ti, Al, K) [6,25,26,27,28] and multivalent anion substitution (e.g., partially replacing PO_4_^3−^ with SiO_4_^4−^) [29] are also emerging recently for higher performances (e.g., lower cost, higher capacities, and improved rate performance with enhanced ion/electron transport) [30].

To be specific, vanadium bronzes (or named vanadium oxide bronzes, abbr. VOBs, M*_x_*V*_y_*O*_z_*, or M*_x_*V_2_O_5_, M = Li, Na, K, Mg, Ca, Zn, NH_4_, Ag, etc.) are typical layered materials with triclinic vanadium–oxygen coordination polyhedral crystal structure and can be identified as vanadium oxides with accommodated electron-donating cations [31,32]. VOBs have raised increasing interest as cathode materials due to their intrinsic large interlayer spacing, mixed valences, high capacity, enhanced electronic conductivity, and structural stability for facilitated intercalation not only in lithium-based batteries but also in non-lithium-based batteries [31,32]. Layered sodium vanadium oxide, e.g., NaV_6_O_15_ with nanoflakes self-assembled microflower structure, can exhibit a plateau of ~2.75 V in the potential window of 4.0–1.5 V, and it can deliver a high capacity of 126 mA h g^−1^ at 100 mA g^−1^ as a cathode material for SIBs, as well as a capacity retention of 87% after 2000 cycles at a high current density of 5 A g^−1^ [33]. While for monoclinic NaVO_3_, the Na^+^ (de)intercalation does not affect the crystal structure and little change occurs for the *a* and *b* lattice parameters (0.13 and 0.19%, respectively), which corresponds to a significantly high specific capacity of 245 mA h g^−1^ in the potential range of 4.7–1.2 V (vs. Na^+^/Na), contributed by the cationic V^5+^/^4+^ and anionic O^−^/O^2−^ redox couples (during discharge process, and the latter redox reaction is partially reversible) [15]; although the possible operating voltage could be higher using density functional theory (DFT) prediction based on the dominant oxygen redox reaction [34].

The vanadium-containing phosphates can exchange even more than one electron per transition metal due to the wide range of oxidation states (+2 to +5) and the diverse polyhedra formed thereof (e.g., tetrahedra, octahedra, pyramids). Two typical redox couples of V^3+^/V^4+^ and V^4+^/V^5+^ (sometimes involving V^3+^/V^2+^ at lower voltages, e.g., below 2 V) have been frequently reported in these various vanadium phosphates. Of which, classic Li_3_V_2_(PO_4_)_3_ and Li-rich layered vanadium mixed-phosphates such as Li_9_V_3_(P_2_O_7_)_3_(PO_4_)_2_ and their derivatives (e.g., Na, Mg-substituted/doped) have been intensively investigated for their high-voltage plateaus (~4.5 V) and high capacities as cathodes of LIBs [35]. NASICON-type Na_3_V_2_(PO_4_)_3_ and its fluorophosphates (e.g., Na_3_V_2_(PO_4_)_2_F_3_) are also intensively studied for SIB cathodes, which both show long-term cycling stability, rate, and high-voltage performances [12].

Fluorophosphates (substituted PO_4_^3−^ with high-electronegativity F^−^) are a novel family of promising cathode materials with even higher voltage output compared to phosphates [7]. Vanadium fluorophosphates with long and high-voltage plateaus and high theoretical capacities have been intensively investigated for LIBs, SIBs, and PIBs, e.g., NaVPO_4_F (theoretical capacity 143 mA h g^−1^) [36], Na_3_V_2_O_2_(PO_4_)_2_F (space group of *I*4/*mmm*, lattice parameters of e.g., *a* = *b* = 6.4958 Å, *c* = 10.61366 Å) [19]. These tetrahedral [PO_4_] and octahedral [VO_5_F]/[VO_4_F_2_] units were interconnected by an O atom in the *ab*-plane (i.e., (002) plane), which can thus be considered as pseudolayered structures with intercalated Na ions on the *ab*-planes [19,36]. The growth of these crystals by the hydrothermal method can be summarized as follows: under appropriate reaction conductions, the positive Na^+^ ions are immediately adsorbed onto the negative molecular clusters, followed by self-assembling along the normal direction of the basal *ab*-plane with the Na ions as pillars to stabilize the structure. The driving force is attributed to the much higher interfacial free energy of the *ab*-plane perpendicular to the *c*-axis than that of the other surfaces; thus, an anisotropic crystal structure such as 1D nanowires or nanorods usually forms in high-chemical-potential surroundings [19]. The possible Na^+^ diffusion paths are mainly between the *ab*-planes from a Na1 site to an adjacent Na1 site, since the activation barriers along the *c*-axis direction are much higher than those in the *ab*-plane (2.248 vs. 0.415 eV, calculated by first principles) [19]. And for 1D-structured materials, the preferred growth along the *c*-axis direction is favorable to the high-rate diffusion of Na^+^ ions since the diffusion paths are perpendicular to the nanowire/nanorod growth direction [19].

### 2.2. Structure, Synthesis, and Electrode Design

As it is known, particle size and intrinsic electrical conductivity affect the diffusion length and electronic/ionic transport; thus, crystal growth and assembly are of great importance. Well-designed nanostructured materials have been considered effective strategies for enhanced intercalation/deintercalation rates due to the shortened ion transport distance and more exposed crystal facets (high surface-to-bulk ratio). A lot of attention has been devoted to the synthesis of nanoscale low-dimensional cathode materials, including 0D nanoparticles [37], 1D nanorods/nanowires [9,38,39], and 2D nanoplates/nanosheets [40], to enhance the rate capability and cycle performance. Nanoparticles herein are classified into 0D materials, and micrometer-sized particles are not intentionally included in this review focusing on low-dimensional materials. To enhance the kinetics of larger alkali-ion (e.g., Na^+^, K^+^) transfer in the cathode, strategies including nanostructuring (decreasing the crystalline size), tuning structures/morphologies, and doping are typically adopted. The synthesis methods mainly include electrospinning (1D, 3D) [41], ball-milling, solid-state reaction (calcination/annealing) or carbothermal reduction (CTR), sol–gel [42], hydrothermal [37,38], etc. Of which, ball-milling and stoichiometric solid-state reactions are intensively used; however, the one-step high-energy ball milling and sol-gel method, together with annealing, i.e., synergistic methods, are promising for future low-cost approaches with high performances. Solid-state reaction and hydrothermal method are typical strategies to realize high-voltage cathode materials with high performance, e.g., solution route followed by solid-state reaction or ball milling followed by solid-state reaction. The high-energy ball milling (i.e., rapid mechanochemical synthesis by a 3D ball-milling machine [43]) may not need further solid-state reaction. Hydrothermal/solvothermal methods will render samples with higher crystallinity and usually no further annealing/calcination is needed.

Beyond the self-assembly featured in sol–gel or hydrothermal methods, some synthesis methods also determine the materials and electrode structures; e.g., electrospinning can produce 1D materials and the 3D aggregates thereof for free-standing/flexible additive-free cathodes of advanced batteries [36,44]. Electrospinning is a facile way to fabricate 1D nanostructures and the crosslinked 3D mat-like framework and is an ideal candidate for cathode materials with sufficient conductivity both for electrons and ions; however, the mass loading, active material ratio, and strength are also of great concern and may not be met for practical applications. And for electrospun 1D materials, active materials inside are in fact not 1D materials but exist in the form of (isolated) nanoparticles dispersing in the conductive backbones (usually carbon-based). Furthermore, some V-based nanoparticles may be in situ coated by carbon layers and/or embedded into 3D conducting networks (e.g., porous graphene network, carbon nanotube framework), which may have superior rate performances up to 100C or even 500C as well as high cycling stability up to even 10,000 cycles [42,45,46]. 

### 2.3. Electrochemical Evaluation

For higher voltage plateaus, a lower vanadium valence (e.g., 3+, 4+) with a specific crystal lattice is usually a critical requirement. Tunnel-structured vanadium phosphates, vanadium fluorophosphates, and layer-structured vanadium oxide bronzes are typical vanadium-containing cathode materials that are showing great promise for ultimately practical application.

Vanadium phosphates and fluorophosphates are intensively investigated as high-voltage (together with flat potential plateaus and very low potential hysteresis) cathode materials for alkali-ion batteries [1,45]. These phosphates/fluorophosphates are showing typical flat potential plateaus. Most of them are generally not only showing high-potential plateaus in 5.0–3.0 V but also low-potential plateaus in 2.0–1.0 V (i.e., big voltage difference, due to V^3+^/V^4+^/V^5+^ redox couple and V^3+^/V^2+^(/V^1+^) redox couple, respectively). Thus they are amphoteric and could be working as either cathodes or anodes, such as Li_3_V_2_(PO_4_)_3_, Na_3_V_2_(PO_4_)_3_ [37], LiVPO_4_F, *β*-LiVP_2_O_7_ [47], *β*-NaVP_2_O_7_ [48], and Li_9_V_3_(P_2_O_7_)_3_(PO_4_)_2_ [49]; symmetric batteries can therefore be configured by using the same material for cathode and anode simultaneously.

For typical polycationic compounds such as Na_3_V_2_(PO_4_)_3_, a very flat potential plateau (~3.4 V vs. Na^+^/Na) is seen, corresponding to the redox reaction between V^3+^/V^4+^ with two Na^+^ extractions/insertions, i.e., a typical two-phase reaction of Na_3_V_2_(PO_4_)_3_ ↔ NaV_2_(PO_4_)_3_, showing an ultralow potential hysteresis of 5 mV [45,50]. These V-based phosphates are usually isostructural to their Fe-based counterparts, and these Na-based phosphates are also isostructural to their Li-based counterparts, which could be prepared by facile electrochemical exchange of Na^+^ for Li^+^ [47]. Although a higher charging voltage will render them with a temporarily elevated voltage/capacity along with a three-phase transition and unexpected solid solution behavior (e.g., a three Li-ions extraction compared to only two Li-ions extraction in the normal two-phase transition for monoclinic *α*-Li_3_V_2_(PO_4_)_3_) [13], it is not stable and unsustainable, i.e., the cycling stability decreases with an increase in upper cut-off voltages due to the irreversible unit cell volume expansion, increased amount of V^5+^ and destruction of carbon layer coating on the surface of active materials [51]. Sodium vanadium pyrophosphates such as Na_7_V_3_(P_2_O_7_)_4_ and Na_2_VOP_2_O_7_ [52] possess a higher redox potential as the vanadium-based cathode of SIBs, an 4V-class electrode. Taking Na_7_V_3_(P_2_O_7_)_4_ as an example, the unique structure with octahedral VO_6_ and connected P_2_O_7_ groups in quasi-layers, as well as the increased inductive effect thereof, contribute to the higher redox potential and output voltage. The electrochemical redox mechanism is also based on the vanadium valence states varying mainly between V^3+^/V^4+^, i.e., Na_7_V_3_(P_2_O_7_)_4_ ↔ Na_4_V_3_(P_2_O_7_)_4_ [12,53,54]. While a similar mixed-phosphate Na_7_V_4_(P_2_O_7_)_4_PO_4_ shares similar electrochemical mechanisms (two-phase transformation of crystal structure during charge/discharge and between V^3+^/V^4+^, and the presence of an intermediate phase endowing it with better kinetics by reducing the lattice mismatch energy to overcome the phase boundary migration), i.e., Na_7_V_4_(P_2_O_7_)_4_PO_4_ ↔ Na_5_V_4_(P_2_O_7_)_4_PO_4_ ↔ Na_3_V_4_(P_2_O_7_)_4_PO_4_, although the voltage plateaus a little bit lower (3.88 V vs. Na^+^/Na, to be specific, 3.87 and 3.89 V for V^4+^/V^3.5+^ and V^3.5+^/V^3+^, respectively) and the capacity somewhat higher [55,56].

**Figure 2:**
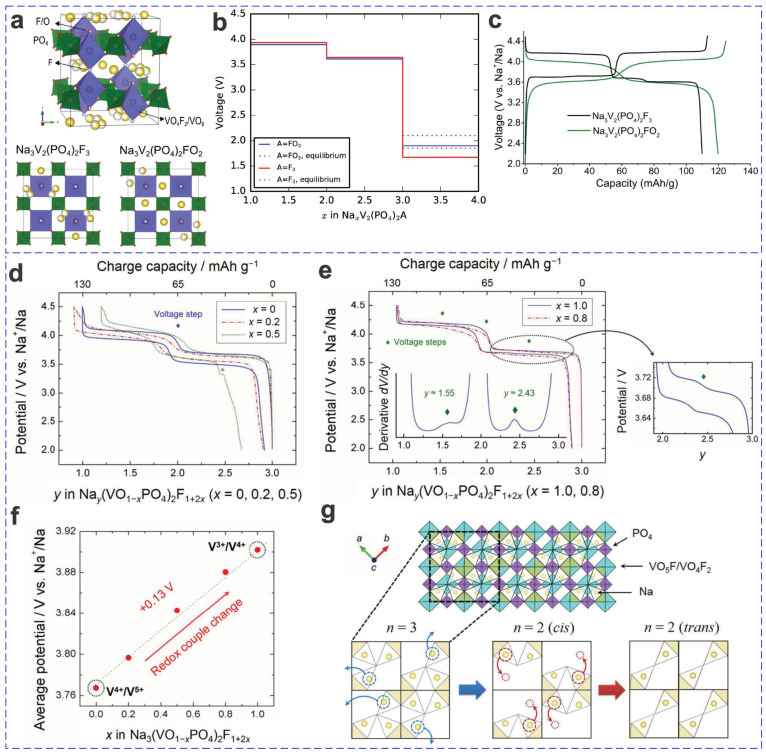
(**a**) Crystal structure of Na_3_V_2_(PO_4_)_2_F_3−2*y*_O_2*y*_ (*y* ≤ 0.2, orthorhombic *Amam* space group, *y* > 0.2, tetragonal *P*4_2_/*mnm* space group, where the significant difference lies in the Na distribution); Top view (*ab*-plane) of the orthorhombic crystal structure (*y* = 0) and the tetragonal crystal structure (*y* = 1). (**b**) The computed voltage curves of Na*_x_*V_2_(PO_4_)_2_F_3−2*y*_O_2*y*_ (1 ≤ *x* ≤ 4; red, *y* = 0, blue, *y* = 1). (**c**) Galvanostatic charge–discharge (GCD) profiles of Na_3_V_2_(PO_4_)_2_F_3−2*y*_O_2*y*_ (*y* = 0 and *y* = 1) at 0.1C rate in the potential window between 2.2 and 4.5 V. Adapted with permission from Ref. [5]. Copyright 2017 Lawrence Berkeley National Lab. Published by WILEY-VCH Verlag GmbH and Co. KGaA, Weinheim, Open Access article under the terms of the Creative Commons Attribution-NonCommercial License. (**d**,**e**) Voltage–composition curves of the Na*_y_*(VO_1−*x*_PO_4_)_2_F_1+2*x*_ electrodes (different *x* value, 0.1C rate). (**f**) Effect of *x* value on the average voltage of Na*_y_*(VO_1−*x*_PO_4_)_2_F_1+2*x*_ electrode, revealing changed redox couple from V^4+^/V^5+^ to V^3+^/V^4+^ when *x* increases from 0 to 1, accompanied by a slight voltage increment of about 0.13 V. (**g**) Phase evolution mechanism referring to Na-vacancy reordering in the Na*_y_*(VO_1−*x*_PO_4_)_2_F_1+2*x*_ (0 ≤ *x* ≤ 1) electrode from the pristine phase (*y* = 3.0) to the intermediate phase (*y* = 2.0) after the Na extraction process. Adapted with permission from Ref. [57]. Copyright 2014 WILEY-VCH Verlag GmbH and Co. KGaA, Weinheim.

The fluorophosphates such as Na_3_V_2_(PO_4_)_2_F_3_ via the substitution of PO_4_^3−^ with F^−^ show even higher operating voltages based on the electrochemical mechanism of V^3+^/V^4+^ redox couple; as a member of the solid solution (oxy)fluorophosphate Na_3_V_2_(PO_4_)_2_F_3−2*y*_O_2*y*_, an increased oxygen content renders it with a slightly lower operating voltage (average ~0.1 V when *y* = 1, via an electrochemical mechanism of V^4+^/V^5+^ redox couple), while the capacity increases slightly (ca. by 10 mA h g^−1^), thus for a slightly higher energy density of 500 vs. 495 W h kg^−1^ [Na_3_V_2_(PO_4_)_2_FO_2_ vs. Na_3_V_2_(PO_4_)_2_F_3_] (Figure 2) [5,57]. The fluorophosphates with higher inductive effect by fluorine usually demonstrate a higher average voltage (ca. 0.3–0.5 V higher) than phosphates, although similar V^3+^/V^4+^ redox transitions occur [58]; furthermore, the V^3+^/V^4+^ transition may show slightly higher voltage than the equivalent V^4+^/V^5+^ redox couple in some cases (e.g., LiVPO_4_F vs. VOPO_4_ of 4.2 vs. 4.0 V). [58]. And for (oxy)fluorophosphate such as Na_3_V_2_O_2_(PO_4_)_2_F, no new phase appeared during the charging–discharging process; only a simple single-phase reaction of electrode material was involved, as can be revealed by the peak shift in ex situ XRD patterns, which relates to the change in lattice parameters; furthermore, the lattice parameter *c* shows a larger rate of change than *a* and *b*, viz., the distortion between the *ab*-planes (i.e., along the *c*-axis direction) is relatively larger than that within the *ab*-plane upon Na^+^ intercalation or deintercalation [19]. Moreover, the volume change of fluorophosphates such as Na_3_V_2_O_2_(PO_4_)_2_F upon full charge is merely 2.79%, which is much smaller than that of Na*_x_*V_2_(PO_4_)_3_ (ca. 8.1%), olivine Na*_x_*FePO_4_ (ca. 17.5%), and mixed-polyanion Na*_x_*Fe_3_(PO_4_)_2_(P_2_O_7_) (ca. 5.1%) [19], also superior to Na_1.5_VPO_4.8_F_0.7_ synthesized by Kang and coworkers in 2013 (once the smallest volume change record, 2.9%) [59].

Some vanadium-based materials, mainly vanadium oxides [60] and vanadium oxide bronzes (VOBs), such as VO_2_ (e.g., nanowires) [10], layered V_2_O_5_ [61,62,63], *γ*-LiV_2_O_5_ [11], monoclinic LiV_3_O_8_ and doped Li_1+*x*_V_3_O_8_ (0 ≤ *x* ≤ 0.2) [64], usually have extraordinarily high capacities (some may be up to over 400 mA h g^−1^ for LIBs or close to 330 mA h g^−1^ for SIBs), although the voltage output is relatively low, e.g., with average voltage output (or long plateau of voltage) near or lower than 3.0 V, which are not focused on here and are cataloged into low-voltage cathode materials. And some similar VOBs (e.g., *ω*-Li_3_V_2_O_5_), however, due to the phase transition (e.g., *γ′*-V_2_O_5_ ↔ *γ*-LiV_2_O_5_ ↔ *ζ*-Li_2_V_2_O_5_ ↔ *ω*-Li_3_V_2_O_5_ based on the reaction process), may be classified as anode materials due to the different alkali ion storage mechanisms and much lower plateaus (wholly or partly falling into the 0–2 V potential window), viz., these materials (V_2_O_5_-based) are binary materials that can both serve as cathodes and anodes [11]. For VOBs, the capacity of these cathode materials usually with mixed valence states may extend up to 200 mA h g^−1^ and beyond, although these materials usually demonstrate relatively low voltage output of ~2 V (an average discharge voltage vs. Li/Li^+^ [32], Mg/Mg^2+^ [65]) compared to high-voltage vanadium-based cathodes of >3.5 V; most of their electrochemical performances, especially the potential output plateaus, are somewhat similar to those of layered vanadium oxides (e.g., *α*-V_2_O_5_), while some specific phases after transition or their corresponding VOBs could show elevated voltage outputs [66]. And for the ammonium ions (NH^4+^) in the specific VOBs, although only a small quality can be reinserted into the layered crystal structure in these batteries (i.e., not fully reversible), they play a crucial role in maintaining the structural stability and improving the electrochemical performances, which is a new insight into the understanding of the intercalation mechanism for these host materials containing ammonium ions [32]. Some other vanadium oxides with high valence, such as LiVO_3_, are also this kind of binary electrodes with a tuned high-/low-voltage percentage via limiting the cut-off voltage [67,68]; while some other transition-metal-based vanadates with high capacities even up to over 1000 mA g^−1^ (e.g., TiO_2_-coated porous FeVO_4_ nanorods [69], porous MoV_2_O_8_ nanosheets [70]) as well as some LVOs (e.g., Li_3_VO_4_ [71,72]) are typical anode materials with major capacity in the potential window of 0–2 V (or even further, 1.0–1.5 V), which are not focused on here. Vanadyl phosphates (polymorphs of VOPO_4_) with layered or tunnel structures can also work as high-voltage cathode materials for alkali-metal batteries; however, initial intercalation/insertion (e.g., prelithiation, presodiation, prepotassiation) is needed for rechargeable alkali-metal-ion batteries [73]. Polyanions such as NaV_3_(PO_4_)_3_ are typical anode materials for SIBs with high stability [74]. Furthermore, it is noteworthy that some layered vanadium oxides with large voltage steps or high initial voltages (>3 V vs. Li^+^/Li, Na^+^/Na, or K^+^/K) are showing specific prospects and thus are also reviewed and commented on here. More detailed information on the parameters of typical vanadium-based high-voltage cathode materials is shown in Table 1.

### 2.4. Battery Assembly and Recycling

Commercial LIBs usually use graphite-based anodes, while for SIBs with larger Na^+^ ions, the hard carbon is in fact the best choice; PIBs, however, could also adopt graphite-based materials such as synthetic graphite as anodes due to the lower electrochemical potential of K compared to Na (−2.93 vs. −2.71, SHE) and the formation of an intermediate compound (KC_8_) during the stage I graphite intercalation, which offer some special advantages over SIBs [80,81]. That is, the V-based SIBs could only choose the hard carbon-based anode, while the V-based LIBs and PIBs could use the hard carbon or conventional graphite-based anodes [82]. And these carbon- or alloy-based full cells possess a higher output voltage than their symmetric counterparts with NASICON-based anodes or other types of anodes such as transition-metal chalcogenides [37,46,50,83]. It should also be mentioned that the V^3+^/V^2+^ transition will endow these vanadium-based compounds with a low potential of ca. 1.4–1.8 V, thus making them a novel class of anode candidates, which could be configured with the higher-potential transitions of V^3+^/V^4+^ and/or V^4+^/V^5+^ (ca. 3.4–4.2 V) for symmetric batteries [6,7].

Electrolyte composition also has a great impact on the performance of cathodes. Taking Na_3_(VOPO_4_)_2_F as an example, the ternary electrolyte compositions of EC/PC/DG (2:2:1) or EC/DEC/DG (2:2:1) + 1.0 M NaClO_4_ can endow them with appreciably high specific capacity (i.e., 105 and 100 mA h g^−1^, respectively, at 0.1C) and high cycling stability, viz., EC/PC shows better performance than EC/DEC, and the addition of diglyme (DG) can further enhance their performances by constructing a more stabilized electrode–electrolyte interface (i.e., solid electrolyte interface, SEI) [84].

**Figure 3:**
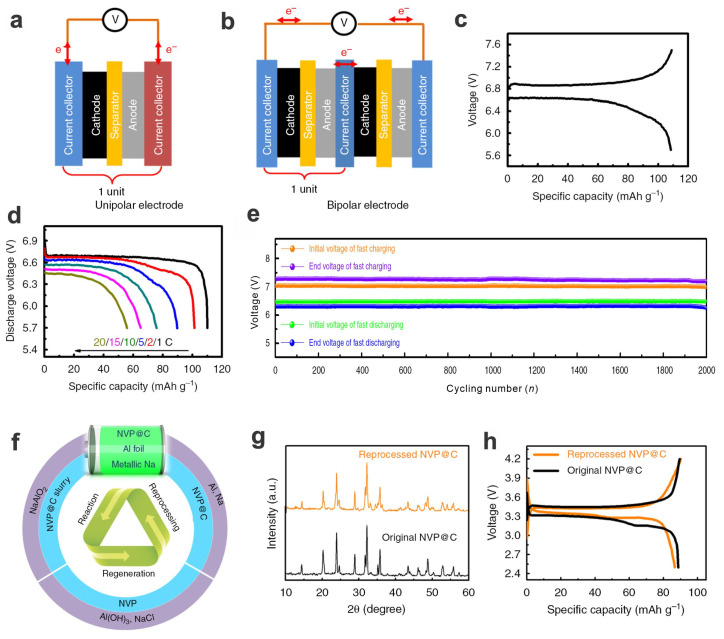
(**a**,**b**) Schematic illustrations of the conventional unipolar electrode design (one-unit cell) and bipolar electrode design (two-unit cell). (**c**–**e**) Typical GCD curves, rate capabilities, and long-term cycling performance of the bipolar SIB cell (NVP@C cathode). (**f**) Schematic illustration of a proposal for the closed-loop utilization of a SIB unit. (**g**,**h**) Characterizations of the recycled NVP@C compared with the original one (XRD spectra and GCD curves at 5C). Adapted with permission from Ref. [85]. Copyright 2019 Springer Nature, Open Access, licensed under a Creative Commons Attribution 4.0 International License.

Due to the increasing environmental concerns about the large-scale application of rechargeable batteries, sustainability, recyclability, and making the best of batteries should be initially considered from the design stage to the end of life. For the electrode design, the bipolar electrode structure (e.g., using Na_3_V_2_(PO_4_)_3_ as a cathode material) with Al foil as the shared current collector will enhance the energy/power densities and long-term cycling stability compared to the traditional design with Cu and Al as unipolar electrodes. Also, most of the solid components can be recycled, e.g., >98.0% on average, with ~100% Na_3_V_2_(PO_4_)_3_ and ~99.1% elemental Al, and these recycled materials could be reutilized to produce new materials with almost undiminished performances (Figure 3) [85,86].

## 3. Applications

### 3.1. Li-Ion Batteries

**Figure 4:**
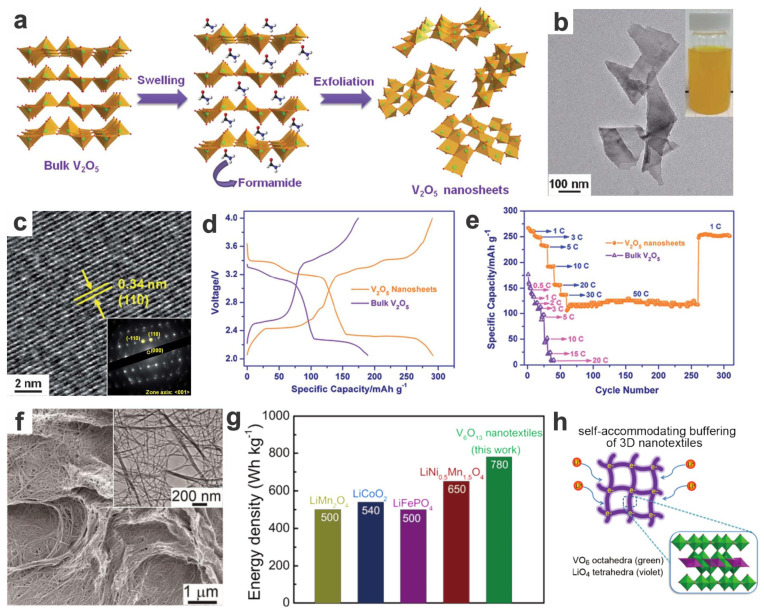
(**a**) Schematic illustration of the simple and scalable liquid exfoliation of layered bulk V_2_O_5_ into (001)-oriented ultrathin few-layer V_2_O_5_ nanosheets and the corresponding (**b**) TEM image (inset: colloidal acetone dispersion) and (**c**) high-resolution TEM image (HRTEM, inset: selected-area electron diffraction pattern, SAED). The electrochemical performances of the as-prepared few-layer V_2_O_5_ nanosheets and bulk V_2_O_5_ as cathodes of LIBs in the voltage window of 4.0–2.05 V: (**d**) initial GCD profiles at 0.2C, and (**e**) rate capabilities. Reproduced with permission from Ref. [88]. Copyright 2013 The Royal Society of Chemistry. (**f**) SEM image of typical 3D V_6_O_13_ nanotextiles prepared by using VOSO_4_ precursor (inset: TEM image). (**g**) Energy density comparison of this 3D nanotextile electrode and typical conventional LIB electrodes. (**h**) Schematic illustration of Li^+^ intercalation in the 3D nanotextile electrode. Adapted with permission from Ref. [89]. Copyright 2015 American Chemical Society.

Vanadium-based cathodes or vanadium-doped/modified cathodes usually show high rate performance, low-temperature features, and even cycling stability. Layer-structured vanadium oxides have high specific capacities [87], however, their potential plateaus are usually not high enough to meet the requirement for high-voltage cathodes in LIBs. Some vanadium oxide-based cathodes, such as well-known V_2_O_5_, may have multiple plateaus (Figure 4a–e) [88], while others, such as mixed-valence V_6_O_15_, could show sloping capacitor-like discharge/charging features [89]. 1D and 2D nanostructures are showing enhanced (rate) capacities and cycling performances compared to the bulk ones [88]. Since these low-dimensional vanadium oxides possess high reversible capacities and rate capabilities, their specific energy/power densities can be extraordinarily high (e.g., 780 W h kg^−1^), far superior to (e.g., 44–56% higher than) those commercial cathodes of LIBs (Figure 4f–h) [89]. However, strictly speaking, vanadium oxides are not typical (Li/Na/K-rich) cathode materials, which need to be preinserted/preintercalated or use alkali metal (e.g., Li, Na, K) or the preinserted carbon as anode, which increases the difficulty of assembly. Nevertheless, it has wide applicability to all sorts of alkali-metal batteries with high-capacity alkali metals as anodes.

**Figure 5:**
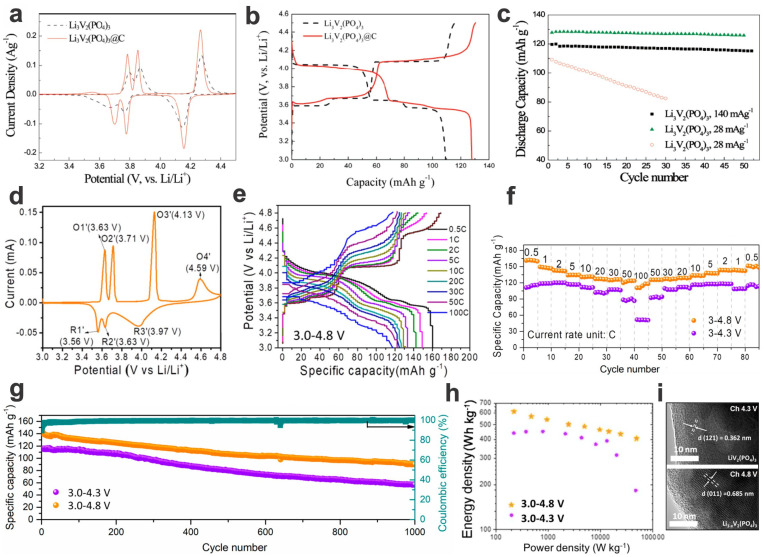
(**a**–**c**) Cyclic voltammograms (CV, 1st cycle), typical GCD curves, and cycling performance of core–shell Li_3_V_2_(PO_4_)_3_@C nanocomposite as LIB cathode compared to pure Li_3_V_2_(PO_4_)_3_. Adapted with permission from Ref. [90]. Copyright 2008, American Chemical Society. (**d**–**g**) CV curves, GCD profiles, rate, and cycling performances of Li_3_V_2_(PO_4_)_3_ cathode of LIB at an extended cut-off voltage of 3.0–4.8 V, as well as (**h**) Ragone plot of the energy density vs. power density and (**i**) ex situ HRTEM images of the Li_3_V_2_(PO_4_)_3_ electrode charged to 4.3 and 4.8 V. Adapted with permission from Ref. [91]. Copyright 2020 American Chemical Society.

In situ carbon coating (hybridization/compositing, or construction of 3D conducting frameworks by using graphene) can greatly improve the electronic conductivity and thus enhance the specific capacity, rate, and cycling performances (for the example of core–shell Li_3_V_2_(PO_4_)_3_@C composites, see Figure 5a–c) [40,63,90]. Charging to a higher voltage can enhance the energy and power densities (taking Li_3_V_2_(PO_4_)_3_ as an example, see Figure 5d–i) [91]; however, the stability of electrolytes and electrodes should be further considered and evaluated.

Ion doping is another way to enhance the rate and cycling performances of lithium vanadium oxides or (fluoro)phosphates (in the Li, V, O, or PO_4_ site, as well as co-doping and interlayer doping) [64]. For example, via K-doping, an optimized Li_0.99_K_0.01_VPO_4_F/C as high-voltage (plateau of 4.2 V vs. Li^+^/Li) cathode for LIBs could show much better rate capabilities (138, 133, 126, and 98 at 0.5, 1, 2, and 10C, respectively) and cycling stability (capacity retention of 96.1% over 125 cycles) compared to pristine LiVPO_4_F/C (capacity retention of 92.2%) or Li_3_V_2_(PO_4_)_3_ [26]. Potassium vanadates (KVO) are another kind of cathode material for LIBs, in which the valence state of vanadium may play a key role. For example, potassium hexavanadate (K_2_V_6_O_16_·*n*H_2_O) nanobelts synthesized by the LPE-IonEx method can deliver a high discharge capacity of 260 mA h g^−1^ in the potential window of 2.0–4.0 V, as well as stable cycling up to 100 cycles at a high current density of 1 A g^−1^. Ex situ measurements demonstrate the electrochemical mechanism of reversible potassium replacement by lithium in the charge/discharge cycle, and the structural stability is mainly attributed to the initial vanadium valence state on the surface and the “fringe free” domains in the KVO nanobelts.

**Figure 6:**
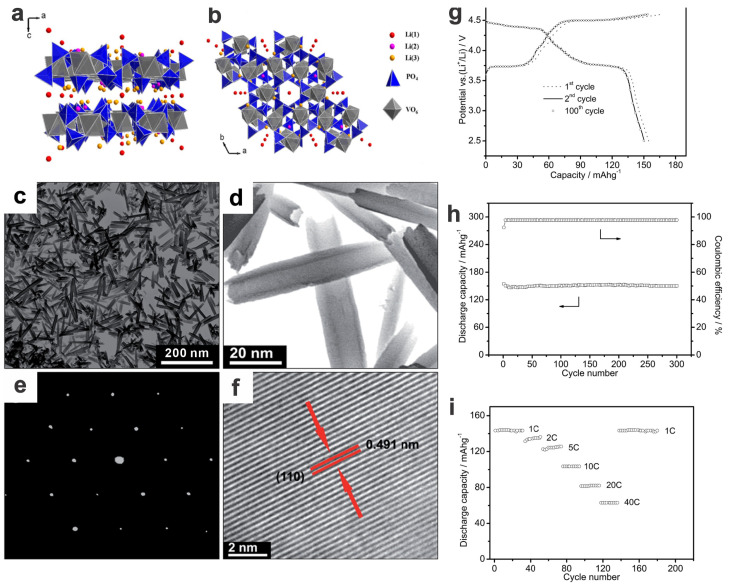
(**a**,**b**) Crystal structure of layered single-phase Li_9_V_3_(P_2_O_7_)_3_(PO_4_)_2_ projected along *b*-axis and *c*-axis. Adapted with permission from Ref. [92]. Copyright 2010 Elsevier Ltd. (**c**,**d**) STEM images, (**e**) SAED pattern, and (**f**) high-resolution STEM image of the as-prepared Li_9_V_3_(P_2_O_7_)_3_(PO_4_)_2_ nanotubes. Electrochemical performances of the Li_9_V_3_(P_2_O_7_)_3_(PO_4_)_2_ nanotubes as cathode material of LIB: (**g**) GCD profiles; (**h**) cycling performance and corresponding coulombic efficiency (CE) at 0.5C; and (**i**) rate performance. Abbr. STEM: Scanning transmission electron microscopy. Adapted with permission from Ref. [75]. Copyright 2015 The Royal Society of Chemistry.

Layered Li-rich mixed-phosphates such as monodiphosphate Li_9_V_3_(P_2_O_7_)_3_(PO_4_)_2_ have extraordinary high voltage output; via tuning the nanostructures, they can be rendered with high cycling stability and rate capability [75,92]. Miao et al. synthesized Li_9_V_3_(P_2_O_7_)_3_(PO_4_)_2_ nanotubes by a simple one-pot molten salt method (580 °C, 10 min under Ar atmosphere), in which the VCl_2_ is injected into the molten salt of LiH_2_PO_4_ and NaNO_3_, followed by the removal of the nitrates with water [75]. The as-prepared nanotubes show enhanced rate capability (143, 123, and 104 mA h g^−1^ at 1, 2, and 10C, respectively, within the cut-off window of 4.6–2.5 V vs. Li^+^/Li; here 1C = 170 mA g^−1^) and demonstrate excellent cycling stability (95.0% capacity retention over 300 cycles at 0.5C, i.e., 147 mA h g^−1^), which are superior to their micrometer-scale counterparts (Figure 6). The shortened ionic diffusion path and enhanced electrical conductivity, due to the small size of robust nanotubes with hollow structures, a thin wall, a high surface area, and high crystallinity, contribute to their superior performances.

### 3.2. Na-Ion Batteries

**Figure 7:**
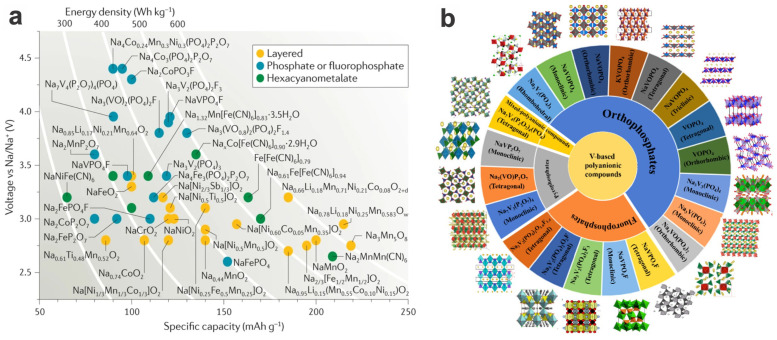
(**a**) Operation voltages vs. specific capacities of SIB cathode materials. Adapted with permission from Ref. [99]. Copyright 2016 Macmillan Publishers Limited. (**b**) Categories of vanadium-based polyanionic compounds as cathode materials for SIBs. Adapted with permission from Ref. [76]. Copyright 2020 Science Press and Dalian Institute of Chemical Physics, Chinese Academy of Sciences. Published by ELSEVIER B.V. and Science Press.

Compared to Li^+^, the Na^+^ ion has a larger radius (1.02 vs. 0.76 Å) and a more positive redox potential (−2.71 vs. −3.04 V), thus the SIBs theoretically/generally show lower power and energy densities compared to LIBs. However, with the significant progress in cathode materials as well as the appropriate anode materials (e.g., hard carbon [93] and other layered materials) in recent years, SIBs are showing great promise to overcome the obstacles for commercial application by using the cathode materials with high rate capability, long cycle life, and high safety, beyond the merits of abundance and low cost [36]. Promising (high-voltage/high-capacity) cathode materials of SIBs (Figure 7a), layered transition-metal oxides (e.g., *γ*-Na_0.96_V_2_O_5_ [94]), Prussian blue/white (including V-based Prussian blue analogs [95,96]), polyanionic compounds, and some arene-based organic molecules/polymers (polyaromatic, conjugated) [97] are intensively investigated, and some of them have been primarily put into scalable production or initial commercialization [1]. Of which, the vanadium-based polyanionic compounds (Figure 7b) with an intrinsic open 3D framework along with additional low-dimensional morphologies are of great interest. These typical examples include orthophosphates [e.g., Na_3_V_2_(PO_4_)_3_, NaVOPO_4_, VOPO_4_], pyrophosphates [e.g., NaVP_2_O_7_, Na_7_V_3_(P_2_O_7_)_4_, Na_2_VOP_2_O_7_], fluorophosphates [e.g., NaVPO_4_F, Na_3_(VO_1−*x*_PO_4_)_2_F_1+2*x*_ (0 ≤ x ≤ 1)], and mixed-phosphates [Na_7_V_4_(P_2_O_7_)_4_PO_4_] [98] (also see details in Table 1 and Table 2), which could be prepared by electrospinning (1D nanofibers and the constructed 3D networks) [41], hydrothermal method [38], sol–gel method [33], and high-temperature solid-state reaction.

**Figure 8:**
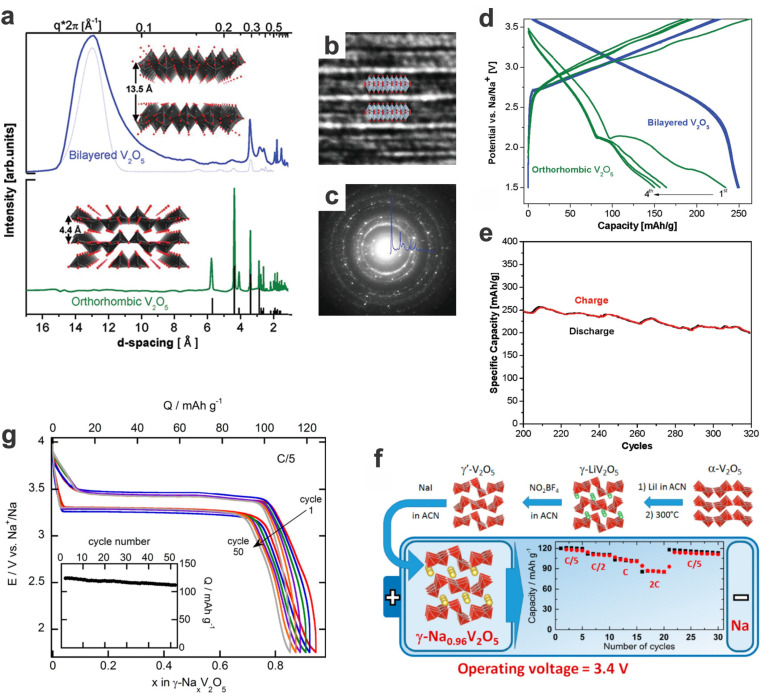
(**a**) X-ray diffraction (XRD) and molecular simulations of bilayered V_2_O_5_ (electrodeposited, annealed in vacuum at 120 °C) and orthorhombic V_2_O_5_ (annealed in oxygen at 500 °C). (**b**,**c**) HRTEM image of bilayered V_2_O_5_ (inset: lattice model) and SAED pattern (inset: XRD pattern). (**d**) Initial four GCD curves of bilayered V_2_O_5_ electrodes compared to orthorhombic V_2_O_5_ electrodes (at 20 mA g^−1^, potential window of 3.8–1.5 V vs. Na^+^/Na). (**e**) Cycling performance of the bilayered V_2_O_5_ as cathode of SIB. Adapted with permission from Ref. [100]. Copyright 2011, American Chemical Society. (**f**) Schematic illustration of the soft chemistry synthesis of *γ*-Na_0.96_V_2_O_5_ from *α*-V_2_O_5_ and its application as a competitive cathode material for SIBs. (**g**) GCD curves *γ*-Na_0.96_V_2_O_5_ (0.2C, potential window of 4.0–1.75 V vs. Na^+^/Na, inset: cycling performance). Adapted with permission from Ref. [94]. Copyright 2018 American Chemical Society.

Bilayered V_2_O_5_ is a specific material with an electrochemically responsive bilayer structure and tunable interlayer distance (13.5 Å), much larger than the classic layered orthorhombic V_2_O_5_ with 4.4 Å. The tailored nanoarchitectures can achieve a theoretical capacity of 250 mA h g^−1^ and good cycling performance with a voltage output of ~3 V, as well as a superior energy density of 760 W h kg^−1^ and a high power density of 1200 W kg^−1^ (Figure 8a–e) [100]. Via the delicate chemical sodiation of similar *γ*′-V_2_O_5_ polymorph at room temperature, sodium vanadium bronze *γ*-Na_0.96_V_2_O_5_ could be obtained, which shows a high capacity (125 mA h g^−1^, corresponding to 0.95 Na^+^ extraction) with a flat discharge voltage plateau of ~3.3 V (vs. Na^+^/Na) in the potential window of 4.0−1.75 V, as well as a high specific capacity of 112 mA g^−1^ at 0.2C maintained after 50 cycles (Figure 8f,g) [94].

**Figure 9:**
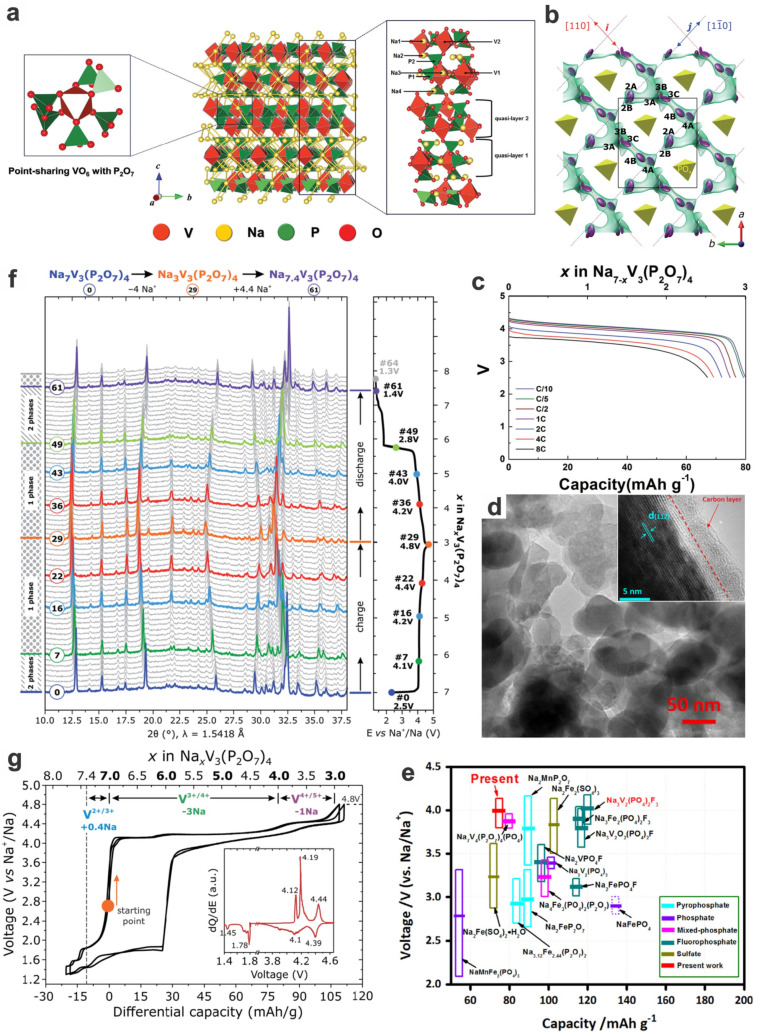
(**a**,**b**) Schematic crystal structure of Na_7_V_3_(P_2_O_7_)_4_ with quasi-layers and 3D/2D Na diffusion pathways. (**c**) Rate capacities of Na_7_V_3_(P_2_O_7_)_4_ in the potential window of 4.35–2.5 V (vs. Na^+^/Na). (**d**) Typical TEM and HRTEM images of an untrafine Na_7_V_3_(P_2_O_7_)_4_ product, and (**e**) the corresponding operating voltage and specific capacity compared to some reported polyanionic materials for SIBs. Note: horizontal bar indicates the average voltage, and vertical band indicates the voltage region. (**f**) In situ XRD patterns of the Na_7_V_3_(P_2_O_7_)_4_ electrode upon charge–discharge in the potential range of 1.3–4.8 V (vs. Na^+^/Na). (**g**) The initial two typical electrochemical cycles (1.3–4.8 V vs. Na^+^/Na), corresponding to the reversible transformation of Na_7.4_V_3_(P_2_O_7_)_4_ ↔ Na_3_V_3_(P_2_O_7_)_4_. (**a**,**c**) Adapted with permission from Ref. [53]. Copyright 2016 WILEY-VCH Verlag GmbH and Co. KGaA, Weinheim. (**b**,**f**,**g**) Adapted with permission from Ref. [12]. Copyright 2020 The Royal Society of Chemistry. (**d**,**e**) Adapted with permission from Ref. [54]. Copyright 2016 Elsevier B.V.

Na_7_V_3_(P_2_O_7_)_4_ is a 4V-class high-voltage cathode of SIBs, despite a lower capacity of ca. 80 mA h g^−1^ [12,53,54]. The open crystal framework synthesized by classical ball-milling and calcination/carbon-coating has a low volume change (~1%), high rate capacities (e.g., 84% retention of the theoretical capacity at 8C), and cycling stability (e.g., 75% capacity retention over 600 cycles at 1C) in the potential window of 4.35–2.5 V (with a relatively flat voltage plateau at ~4.13 V) (Figure 9a–c) [53]. In addition, the V^3+^/V^4+^ transformation mechanism, a small portion of V^4+^/V^5+^ transformation (up to an average of 4.33 V when charged to 4.8 V) also occurs at higher charge/discharge stages for possible higher capacities (not fully reversible) (Figure 9d–g) [12]. However, the adverse effect of overcharging (more than normal electron per V ion) on structural and cycling stability as well as probable electrolyte decomposition should be further investigated for improvement of electrochemical stability or to balance the capacity enhancement against the adverse effect [12,101].

However, mixed-phosphates such as Na_7_V_4_(P_2_O_7_)_4_(PO_4_) (e.g., nanorods, microparticles) are showing slightly lower voltages (e.g., potential plateau of ~3.88 V vs. Na^+^/Na), although the capacities may somewhat increase (e.g., ca. 90 mA h g^−1^) [55,56]. A relatively high capacity retention rate of 78% could be achieved over 1000 cycles [55].

As the most typical NASICON-type vanadium phosphate, Na_3_V_2_(PO_4_)_3_ (NVP) possesses a high operating voltage of ~3.4 V (vs. Na^+^/Na) and a moderate specific capacity of 118 mA h g^−1^ and an energy density of ~400 W h kg^−1^ (theoretically); the F^−^/Cl^−^ substitution will further enhance the operating voltage and the energy density thereof [29,42]. By regulating the substitution ratio or F/O ratio, the redox potential and capacities of Na_3_V_2_(PO)_2_O_2–2*x*_F_1+*x*_ (0 ≤ x ≤ 1) could be tuned, and these with diminished inductive effects could boost the electrochemical performances [29]. The Na_3_V_2_(PO_4_)_2_O_2_F (NVPOF), for example, could provide higher potential plateaus (ca. 4.0/3.6 V vs. Na^+^/Na due to the V^4+^/V^5+^ redox couple) and a high theoretical capacity of 130 mA h g^−1^ and an energy density of ~500 W h kg^−1^ [29]. Another family of Na_3_V^III^_2−*y*_(V^IV^O)*_y_*(PO_4_)_2_F_3−*y*_ (NVPFO*_y_*) (0 ≤ *y* ≤ 2) with mixed-valence states of vanadium (+3 and +4) also shows similar performances, while an optimized oxygen content (or V^3+^/V^4+^ initial ratio, e.g., *y* = 1.35) can endow the cathode with the lowest electrical resistivity, best rate capabilities, and long-term cycling stability [102,103]. The particle morphology may have a significant impact not only on the capacities and rate capabilities but also on the cycling performances; the nanoparticles possess the best performances, followed by 3D flower-like structures, cylindrical aggregates, and nanoflakes. It should be mentioned that sometimes failing to obtain a pure phase does not necessarily mean a failure, viz., a mixture of multiphase vanadium compounds consisting of Na_3_V_2_(PO_4_)_2_F_3_, Na_3_V_2_(PO_4_)_3_, V_2_O_3_, and Na_3_VF_6_ following a carbon thermal reduction/solid-state reaction method does not have to be such a disaster, and they could settle into complementary roles for comparable performances [104]. Another kind of layered NaVOPO_4_ plates (stacked) synthesized by refluxing, hydrothermal, and ball milling with Ketjen black could demonstrate a high voltage plateau of ~3.5 V (vs. Na^+^/Na, slightly sloping), a high specific capacity of 144 mA h g^−1^ (close to the theoretical capacity of 145 mA h g^−1^ based on 1 e^−^ transfer) at 0.05C, a high energy density of 504 W h kg^−1^, and a fair long-term cycling stability over 1000 cycles (capacity retention of 67%) [105].

**Figure 10:**
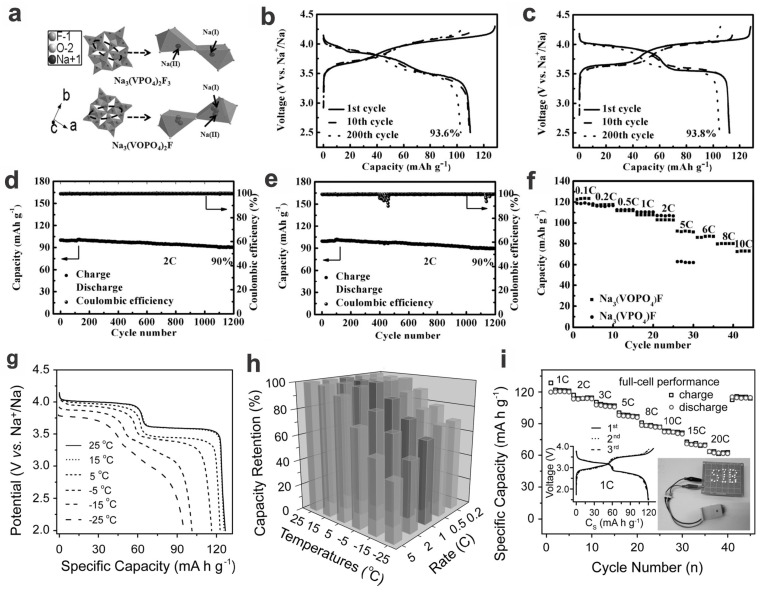
(**a**) Schematic illustration of the coordination polyhedron around Na^+^ ions in Na_3_(VPO_4_)_2_F_3_ and Na_3_(VOPO_4_)_2_F. Electrochemical performances of solvothermally low-temperature-synthesized Na_3_(VPO_4_)_2_F_3_ and Na_3_(VOPO_4_)_2_F nanoparticle electrodes: (**b**,**c**) GCD curves at 0.2C; (**d**,**e**) cycling performance and coulombic efficiency at 2C; and (**f**) rate capabilities. Adapted with permission from Ref. [106]. Copyright 2015 Wiley-VCH Verlag GmbH and Co. KGaA, Weinheim. Electrochemical properties of hydrothermally synthesized Na_3_V_2_(PO_4_)_2_O_2_F nano-tetraprisms (NVPF-NTP) in half cells and full cells: (**g**) galvanostatic discharge curves at 0.2C in the temperature range from 25 to −25 °C, (**h**) the corresponding capacity retentions in the temperature range of 25 to −25 °C at various rates of 0.2–5C, and (**i**) the performance of NVPF-NTP||Sb-CNT full cell (rate capabilities, insets: GCD curves and a full cell prototype powering 33 LED bulbs). Adapted with permission from Ref. [14]. Copyright 2017 WILEY-VCH Verlag GmbH and Co. KGaA, Weinheim.

Compared with ball-milling strategies, the hydrothermal/solvothermal methods could better regulate the nano-/microstructures of the products. For example, NVP and Na_3_(VOPO_4_)_2_F (NVOPF) could be deliberately synthesized and are showing similar capacities and cycling performances, although NVOPF is exhibiting better high-rate capacities (up to 10C rate) (Figure 10a–f) [106]. The Na_3_V_2_(PO_4_)_2_O_2_F nano-tetraprisms (NVPF-NTP) synthesized by Guo et al. have two typical high-voltage plateaus at 4.0 and 3.6 V (vs. Na^+^/Na), and are showing a high specific capacity of 127.8 mA h g^−1^ at 0.1C (close to the theoretical value of 130 mA h g^−1^) and an energy density of up to 486 W h kg^−1^, higher than most of the other cathode materials previously published for SIBs. The low strain (~2.56% volumetric change) and outstanding Na^+^ transport kinetics in the insertion/extraction processes endow them with superior low-temperature performance (down to −25 °C, a specific capacity of 96.1 mA h g^−1^ at 0.2C, 76.4% capacity retention compared to that at room temperature, i.e., 25 °C), and excellent high-rate capabilities, not only in the half cells but also in the full cells configured with a metalloid Sb-based anode (Figure 10g–i) [14].

**Figure 11:**
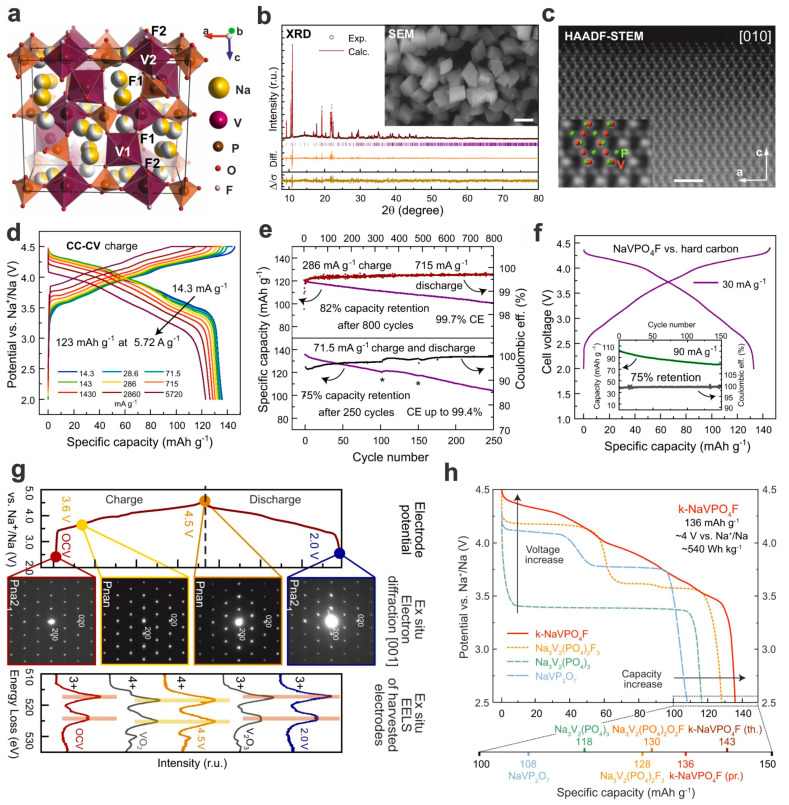
Composition, crystal structure, and electrochemical performances of KTiOPO_4_-type (abbr. KTP or k-type) NaVPO_4_F. (**a**) Schematic illustration of the crystal structure; (**b**) XRD data (inset: SEM image, 1 μm scale bar); (**c**) [010] HAADF-STEM image (2 nm scale bar); (**d**) GCD curves at different rates in the potential range of 2.0–4.5 V (vs. Na^+^/Na), and (**e**) cycling performances at high rates for 800 cycles and low rates for 250 cycles (* marks electrolyte refreshment). (**f**) GCD curves for the NaVPO_4_F||HC full cell (with presodiated hard carbon anode, inset: cycling performance). (**g**) Structural evolution and charge compensation mechanism analysis of NaVPO_4_F during cycling by the corresponding GCD profile in the NaVPO_4_F||Na cell, [001] ED patterns, and ex situ EELS spectra (inserted V_2_O_3_ and VO_2_ spectra are given for reference), showing the reversible changes of phase transition and vanadium oxidation state. (**h**) Potential vs. specific capacity profiles for k-NaVPO_4_F (th.: theoretical, pr.: experimental) compared with other typical vanadium-based polyanion cathodes of SIBs. Abbr. HAADF-STEM: high-angle annular dark-field scanning transmission electron microscopy; ED: electron diffraction; EELS: electron energy-loss spectra. Adapted with permission from Ref. [107]. Copyright 2022 The Author(s). Published by Springer Nature, Open Access licensed under a Creative Commons Attribution 4.0 International License.

Fedotov and coauthors developed a vanadium-based polyanion cathode material (k-NaVPO_4_F) with high voltage output (around 4.0 V) as well as enhanced specific capacity up to 136 mA h g^−1^ at 14.3 mA g^−1^ and superior rate capacity of 123 mA h g^−1^ at 5.7 A g^−1^ [107]. The incorporation of NaVPO_4_F composition with KTiOPO_4_-type framework by a relatively low-temperature ion-exchange synthesis method (190 °C) benefits performances as well as promising practical applications. When configured with a Na metal anode and a common NaPF_6_-based organic electrolyte, it could surpass typical vanadium-based polyanion cathode materials in both voltage output and specific capacity. The Na-metal battery shows a capacity retention of 75% after 250 cycles at a low current density of 71.5 mA g^−1^ and could reach an 82% capacity retention after 800 cycles at a higher current density (charge at 286 mA h g^−1^ and discharge at 715 mA h g^−1^). The Na-ion battery with hard carbon as anode demonstrates a slightly lower voltage output in the potential range of 2.0–4.4 V and shows a comparable specific capacity and cycling performance at low rates, e.g., 75% capacity retention at a current density of 90 mA g^−1^. Via ex situ analysis, a reversible *Pna*2_1_-to-*Pnan* transition and a V^3+^ ↔ V^4+^ transition were revealed during the extraction/insertion of Na^+^ ions (Figure 11). 

**Figure 12:**
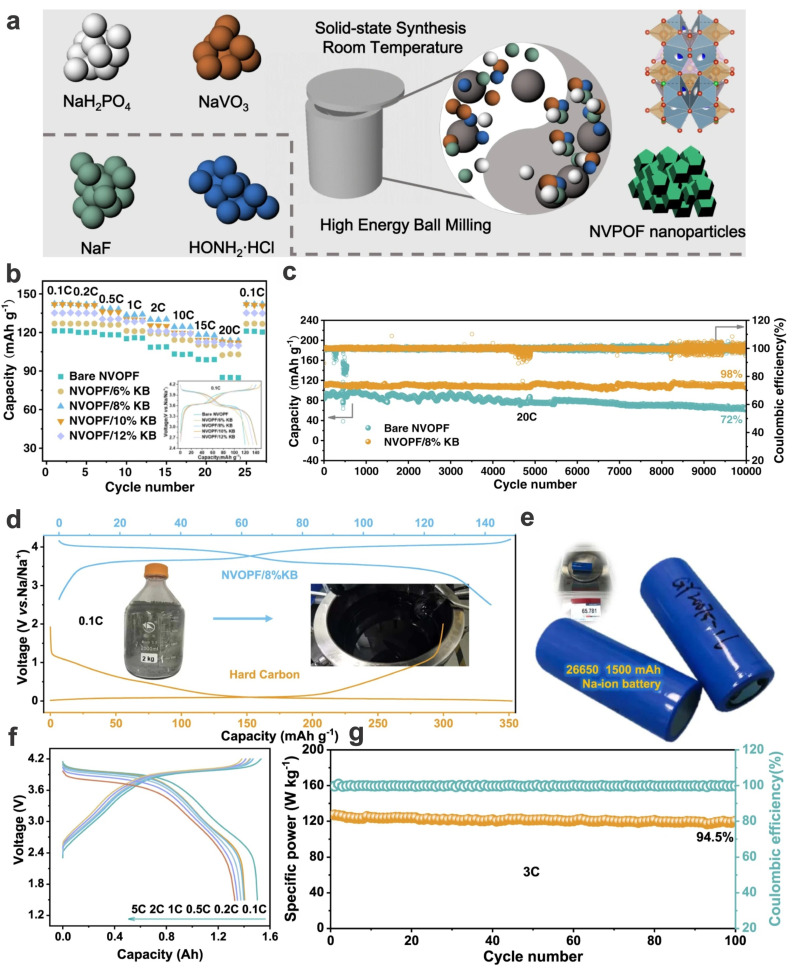
(**a**) Schematic illustration of the mechanochemical synthesis of Na_3_(VOPO_4_)_2_F (NVOPF) nanoparticles starting from precursor NaVO_3_. (**b**) Rate capabilities of carbon-coated NVOPF/Ketjen black (NVOPF/KB) nanocomposites with different KB contents (inset: corresponding GCD curves at 0.1C). (**c**) Ultralong cycling performance of NVOPF/8% KB and bare NVOPF electrodes at 20C. Illustration for large-scale production of the NVOPF/KB composite and the prototype 26,650 full-cell performance: (**d**) GCD profiles of NVOPF/KB cathode and commercial hard carbon anode (inset: the kilogram-scale NVOPF/8% KB product and the preparation of slurry); (**e**) Photograph of a 26,650 prototype cell (weight: ~66 g); (**f**,**g**) Rate capabilities and cycling performance of the 26,650 Na-ion battery. Adapted with permission from Ref. [43]. Copyright 2021 The Author(s). Published by Springer Nature, Open Access licensed under a Creative Commons Attribution 4.0 International License.

Shen et al. prepared a carbon-coated Na_3_(VOPO_4_)_2_F nanocomposite with a rapid mechanochemical synthesis route (Figure 12a) [43]. The optimized NVOPF/8%KB (with an optimized 8% conductive agent of Ketjen black) demonstrated a superior specific capacity of 142.2 mA h g^−1^ in the range of 4.2–2.5 V (featured with two flat plateaus of 4.0 and 3.6 V), even higher than the theoretical value of 130 mA h g^−1^, and an outstanding high rate capacity of 113.2 mA h g^−1^ was achieved at 20C, compared to 84.8 mA h g^−1^ of bare NVOPF without carbon coating. With a relatively high loading of active materials (6 mg cm^−2^), the as-prepared cathode NVOPF/8%KB can maintain an excellent specific capacity of 110.5 mA h g^−1^ after superlong 10,000 cycles at a high C-rate of 20C, corresponding to a superior capacity retention of 98% and a decay rate of merely 0.0002 mA h g^−1^ per cycle (Figure 12b,c). When configured with typical hard carbon as anode, the full cell of NVOPF/8%KB||HC could show a high capacity of 87, 74, 67, 58, and 49 mA h g^−1^ at 0.5, 1, 2, 5, and 10C, respectively, and a capacity retention of 90.7% was obtained after 100 cycles at 2C. A kilogram-scale synthesis could be realized, and the 26650-protype cells were assembled for desirable performances (Figure 12d–g), which is an inspiring step in the direction of industrial application of vanadium fluorophosphates for promising Na-ion batteries.

**Figure 13:**
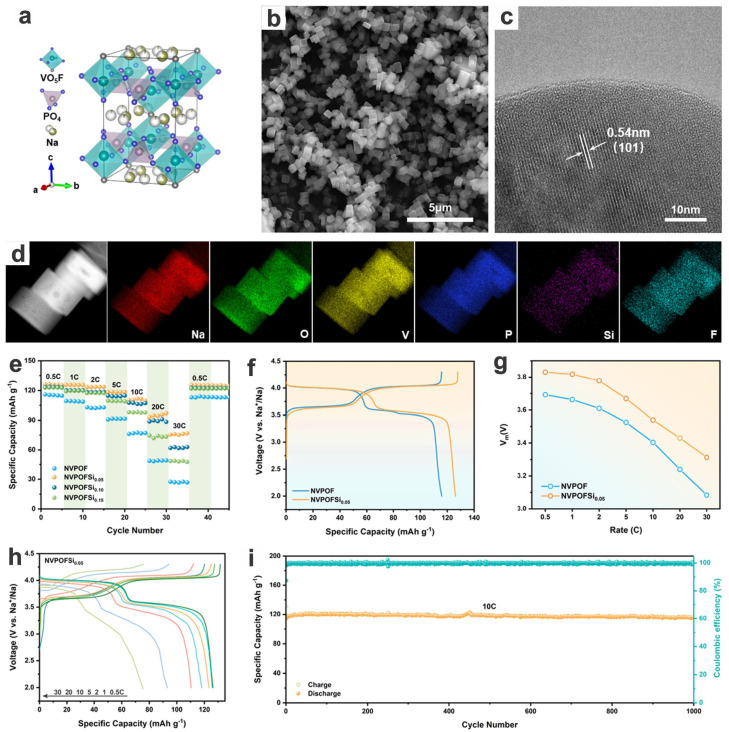
(**a**) Crystal structure of Na_3_V_2_(PO_4_)_2_O_2_F (NVPOF). (**b**–**d**) SEM image, HRTEM image, and HAADF-STEM image and corresponding elemental mapping of Na_3_V_2_(PO_4_)_1.95_(SiO_4_)_0.05_O_2_F (NVPOFSi_0.05_) material. Electrochemical performances of NVPOF and NVPOFSi_0.05_ materials: (**e**) rate capabilities of NVPOF and three SiO_4_^−^-substituted NVPOFSi*_x_* cathodes; (**f**) comparison of the first GCD profiles at 0.5C; (**g**) mean discharge voltage (*V*_m_) at various rates; (**h**) GCD profiles of NVPOFSi_0.05_ cathode at different rates; and (**i**) cycling performance of NVPOFSi_0.05_ cathode at 10C. Adapted with permission from Ref. [29]. Copyright 2023 Elsevier Ltd.

**Figure 14:**
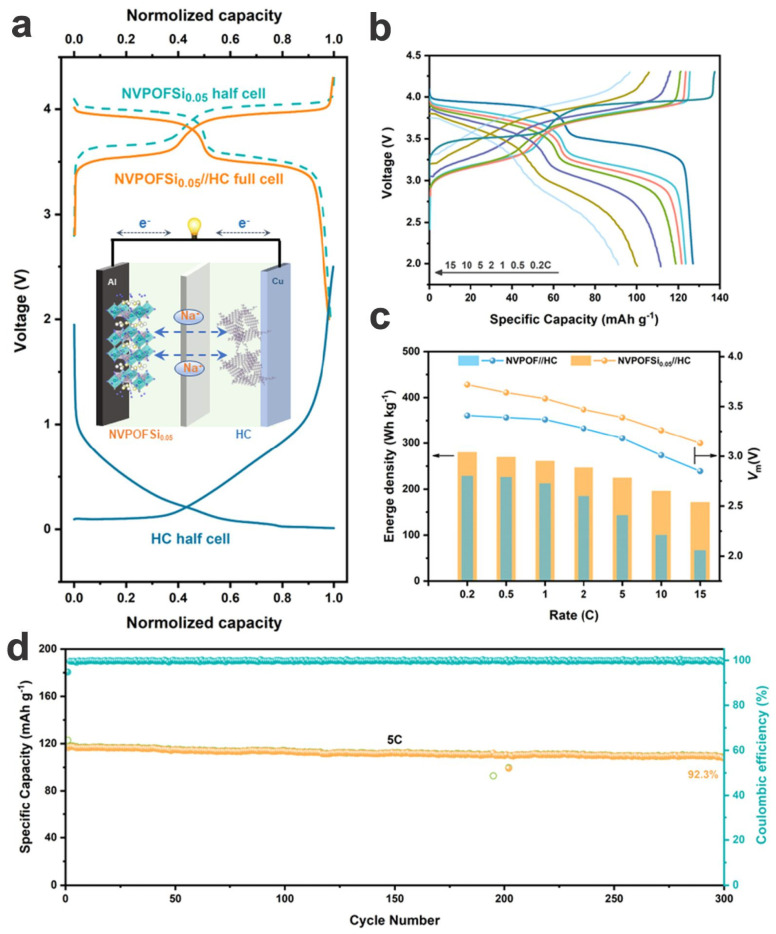
Electrochemical performances of NVPOFSi_0.05_||HC-based Na-ion full cells. (**a**) GCD profiles of NVPOFSi_0.05_||HC full cell (inset: schematic illustration), and the contrastive profiles of NVPOFSi_0.05_ and HC electrodes in half cells. (**b**) GCD profiles of NVPOFSi_0.05_||HC full cell. (**c**) Energy density and *V*_m_ of NVPOFSi_0.05_||HC full cell, compared to NVPOF||HC full cell. (**d**) Cycling performance of NVPOFSi_0.05_||HC full cell at 5C rate. Adapted with permission from Ref. [29]. Copyright 2023 Elsevier Ltd.

The performances may be improved by further modifications, such as doping/substitution and compositing. Zhou et al. synthesized a kind of anion SiO_4_^4−^-substituted Na_3_V_2_(PO_4_)_2_O_2_F nanocubes, i.e., Na_3_V_2_(PO_4_)_1.95_(SiO_4_)_0.05_O_2_F (NVPOFSi_0.05_), via partial replacement to further improve the intrinsically low electronic conductivity (Figure 13a–d) [29]. Experimental and theoretical calculation support the assumption as well as the broadened ionic transport channels, which synergistically bring about a significant enhancement in specific capacity (126 mA h g^−1^ at 0.5C compared to 116 mA h g^−1^ of NVPOF), high-rate capabilities up to 75.5 mA h g^−1^ at 30C (126, 118, and 110 mA h g^−1^ at 1, 5, and 10C, respectively), and long-term cycling stability with almost no capacity loss over 1000 cycles at 10C (Figure 13e–i). Due to the enhanced capacities and rate capability, the full cell configured with a hard carbon anode shows a high energy density of 280 W h kg^−1^ and long-term cyclability with 92.3% capacity retention over 300 cycles at a high rate of 5C (of which the cathode is contributing a preeminent rate capacity of 91.2 mA h g^−1^ at 15C) (Figure 14). The anion substitution gives new insight into the mechanism of polyanionic crystal engineering and opens up new possibilities for the design of high-performance cathode materials.

**Figure 15:**
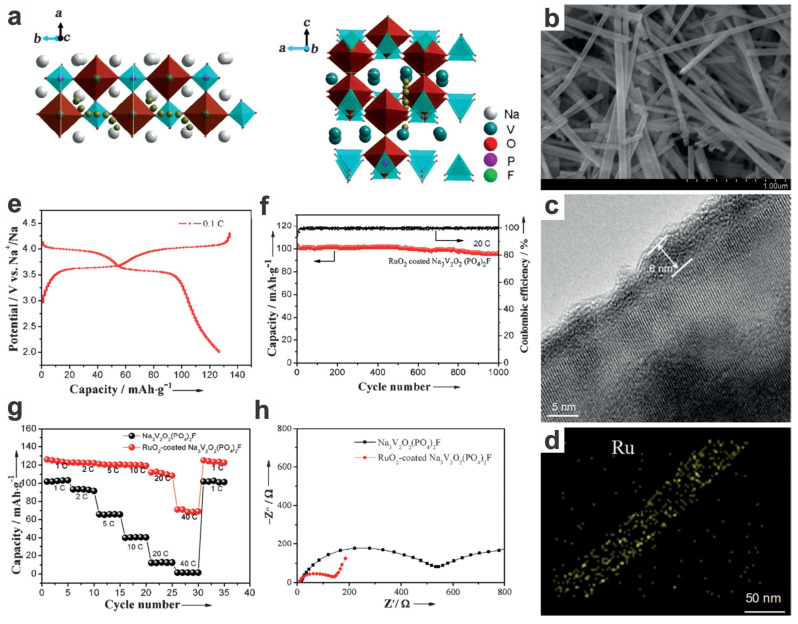
(**a**) Schematic illustration of the crystal structure of Na_3_V_2_O_2_(PO_4_)_2_F in the *ab*-plane and *ac*-plane, as well as the diffusion paths. (**b**) SEM image of Na_3_V_2_O_2_(PO_4_)_2_F nanowires. (**c**) HRTEM image of RuO_2_-coated Na_3_V_2_O_2_(PO_4_)_2_F nanowires (RuO_2_ layer thickness ~6 nm), and (**d**) corresponding elemental mapping of Ru. Electrochemical performances of RuO_2_-coated Na_3_V_2_O_2_(PO_4_)_2_F nanowires as cathode material for SIBs, compared to pristine Na_3_V_2_O_2_(PO_4_)_2_F nanowires (without coating): (**e**) GCD profiles at 0.1C in the voltage window of 4.3–2.5 V (vs. Na^+^/Na), (**f**) long-term cycling performance at 20C, (**g**) rate capacities, and (**h**) Nyquist plots. Adapted with permission from Ref. [19]. Copyright 2015 Wiley-VCH Verlag GmbH and Co. KGaA, Weinheim.

To further improve the rate capability and long-term stability compared to pristine Na_3_V_2_O_2_(PO_4_)_2_F, Peng et al. prepared oxygen-deficient RuO_2_-coated Na_3_V_2_O_2_(PO_4_)_2_F nanowires (NVOPF, ca. 50 nm in diameter) via microemulsion-mediated hydrothermal synthesis (180 °C, 24 h, pH = 2–3, surfactant CTAB, precursors of NH_4_VO_3_, NH_4_H_2_PO_4_, NaF, and H_2_C_2_O_4_, liquid phase: water/cyclohexane/*n*-pentanol) [19]. The as-prepared RuO_2_-coated NVOPF nanowires (core–shell structure, optimal shell thickness of ~6 nm) with promoted electrical conductivity can deliver an enhanced reversible capability up to 120 mA h g^−1^ at 1C, while the pristine uncoated NVOPF nanowires merely show a capacity of 101 mA h g^−1^ at 1C in the voltage window of 4.3–2.5 V. And an outstanding high rate capacity of 95 mA h g^−1^ at 20C can be maintained after 1000 cycles, with a capacity retention of ~95% and gradual decaying of only ca. 0.005% per cycle (Figure 15). The high rate performance is attributed to the facilitated Na^+^ diffusion along the preferably grown (002) plane, which is disclosed by the first principles computation. Via tuning the pH value of the solution, the micromorphology of the NVOPF could be controlled into others, e.g., nanorods and nanoplates (at higher pH values of 3–5, 5–7, vs. 2–3 for nanowires), although they are showing lower reversible capacities; the level of generality and mechanism need further investigation. Some other research on 3D architectures of Na_3_V_2_O_2_(PO_4_)_2_F nanocubes@carbon/graphene [108] and crosslinked graphene-caged Na_3_V_2_(PO_4_)_2_F_3_ microcubes (NVPF@rGO) [109] composites is also showing improved rate capabilities and reversible capacities.

**Figure 16:**
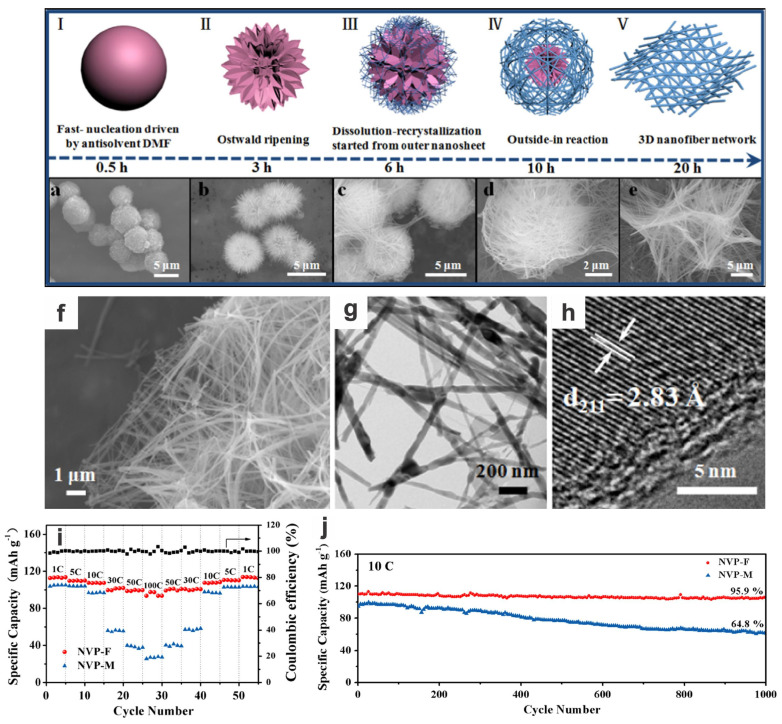
(**a**–**e**) Schematic illustration of a time-dependent solvothermal reaction for 3D Na_3_V_2_(PO_4_)_3_ nanofiber network (NVP-F), i.e., a self-sacrificed evolution from microsphere to nanofiber network (microsphere → nanosheet-assembled microflower → nanofiber network via successive mechanisms of nucleation and growth, Ostwald ripening, dissolution–recrystallization). (**f**) SEM image of NVP-F after annealing, and (**g**,**h**) the corresponding TEM and HRTEM images. (**i**,**j**) Rate capabilities and cycling performance (at 10C) of the NVP-F as high-rate cathode of SIBs, compared with NVP-M (microflower) electrode. Adapted with permission from Ref. [38]. Copyright 2016 Elsevier Ltd.

Low-dimensional V-based materials may incorporate conducting matrices and assembly into 3D or hierarchical composite structures for better rate and cycling performances due to the enhanced apparent Na^+^ diffusion coefficient and high electrical conductivity within the bulk electrode. In situ carbon coating is a facile and most effective strategy amongst so-called carbon engineering [37,110]. For example, in situ amorphous carbon-coated Na_3_V_2_(PO_4_)_3_ shows an excellent rate capability (114 mA h g^−1^ at 1C, and 92% and 54% are retained at 10C and 40C, respectively) and cycling stability (50% retention of initial capacity after 30,000 cycles at 40C); and further construction of amorphous carbon-coated Na_3_V_2_(PO_4_)_3_ in a 3D porous graphene network can deliver an outstanding rate capability of 86 mA h g^−1^ at 100C and an excellent cycling stability of 64% retention after 10,000 cycles at 100C (ultralow capacity decay per cycle) [45]. These high rate performances usually correspond to high energy/power densities, e.g., a superior gravimetric energy density of 384 W h kg^−1^ (at a power density of 0.4 kW kg^−1^) and a high power density of 33 kW kg^−1^ (36 s charge–discharge rate, maintaining 62% of the energy density, i.e., 239 W h kg^−1^), better than normal LIBs. However, the inappropriate matching with anodes may deteriorate the performance of the full cells and their practical application. Yu and coworkers synthesized a kind of porous 1D Na_3_V_2_(PO_4_)_3_@C nanowires via surfactant-assisted hydrothermal method followed by post-heating treatment, in which the nanosized carbon-coated-NVP nanoparticles are embedded in the 1D porous carbon nanowires to improve the intrinsically poor electric conductivity of NVP and the ion-transport and electrolyte access are also enhanced [8]. As a cathode of SIBs, it shows the lowest polarization and can exhibit excellent rate capabilities (84, 77, and 62 mA h g^−1^ at 30, 40, and 60C, respectively) and a high cycling stability with a high 96.6 mA h g^−1^ after 1000 cycles at 1C, far superior to these of micrometer-sized pure NVP or NVP/C samples synthesized without surfactant assistance/carbon source [8,37]. The only flat plateau around 3.4 V between the cut-off window of 2.3–3.9 V corresponds to the V^3+^/V^4+^ redox couple. 1D NVP cathode materials could also be synthesized via a hydrothermal approach based on a possible self-sacrificed morphological evolution mechanism, and the quasi-3D NVP nanofiber network with improved electrical transport merits could as well demonstrate outstanding rate and cycling performances, superior to their microflower-structured counterparts (Figure 16) [38].

**Figure 17:**
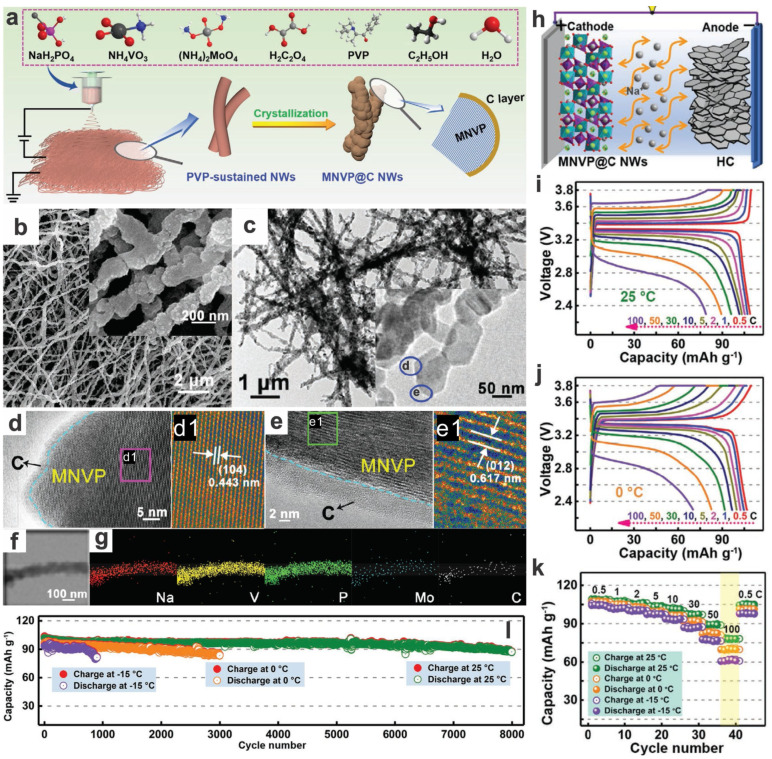
(**a**) Schematic illustration of the preparation of Mo-doped Na_3_V_2_(PO_4_)_3_ nanowires with in situ coated carbon nanoshell (MNVP@C NWs) via electrospinning and annealing. (**b**–**g**) SEM images, TEM images, HRTEM images, STEM image and corresponding elemental mapping of the as-prepared MNVP@C NWs. Electrochemical evaluation of the Na-ion full cells of MNVP@C NWs||presodiated HC: (**h**) schematic illustration of the full cell, (**i**,**j**) GCD profiles at 25 and 0 °C with rates ranging from 0.5 to 100C, (**k**) rate capabilities at −15, 0, and 25 °C; and (**l**) the corresponding long-term cycle stability. Adapted with permission from Ref. [113]. Copyright 2021 Wiley-VCH GmbH.

1D Na_3_V_2_(PO_4_)_3_@C nanowires can also be synthesized via the classic, general electrospinning method [111,112]. Their reversible capacity and rate capabilities could be tuned by the pyrolysis/calcination conditions, e.g., 800 °C, 10 h, 2.5 °C min^−1^ (heating rate) is an optimized one for the polyvinylpyrrolidone (PVP K90)/NVP system to reach the ideal discharge platform of near 3.4 V, close to the theoretical capacity (114 mA h g^−1^ at 0.05C), and maintaining a high capacity (78 mA h g^−1^ at 10C) as well as high cycling stability and coulombic efficiency [112]. Yuan group further fabricated Mo-doped Na_3_V_2_(PO_4_)_3_@C nanowires via electrospinning, followed by pyrolysis and annealing (Figure 17a–g) [113]. The as-prepared Mo-doped NVP@C nanowires show high-rate and long-term cycling stability with wide temperature tolerance (i.e., from −25 to 55 °C), due to their unique compositional/structural merits (in situ coated Mo-doped 100 nm NVP nanoparticles connecting to 1D nanowires and the assembled 3D networks thereof). Mo-doping and in situ carbon coating enhanced the electronic/ionic transport and structural stability/tolerance. The as-prepared sample shows a high initial discharge capacity of 116.8 mA h g^−1^ in the potential window of 4.0–2.5 V, close to its theoretical capacity of 117 mA h g^−1^ (NVP), higher than control samples without doping (114 mA h g^−1^) or bulk ones (110 mA h g^−1^). And it also demonstrates outstanding rate capacities (99, 93, and 80 mA h g^−1^ at 50, 100, and 150C, respectively), compared to undoped (87, 78, and 62 mA h g^−1^ at the same rates) and bulk (17 mA h g^−1^ at 30C) counterparts. The capacity retention is also competent, at 95.3% at 1C and 93.0% at 5C, i.e., remaining capacities of 114.8 and 101.4 mA h g^−1^. The pouch-type full cell configured with a hard carbon anode demonstrates a high energy density of 262 W h kg^−1^ (material-level, 25 °C), a high reversible capacity of 87.3 mA h g^−1^ at 5C after 8000 cycles (85.7% retention of the initial capacity at 25 °C), and A good low-temperature tolerance (86.5% after 3000 cycles at 0 °C and 87.0% after 1000 cycles at −15 °C) (Figure 17h–l); furthermore, the cathode can also work as a cathode for hybrid Li/Na-ion batteries (HLNIBs, balancing the merits of LIBs and SIBs), and can deliver a high-rate, temperature-tolerance, long-duration cycling lifespan [113]. Moreover, the adoption of alloy-based anodes such as Se (e.g., in the form of 3D Se/graphene composite) can also show a superior cycling lifespan (15,000 cycles with a capacity retention of 86.3%, i.e., working life of >60 years under the conditions of 80% capacity retention and charge/discharge once a day) and a temperature tolerance down to −25 °C (e.g., with a capacity retention > 75% after 1000 cycles at 0.4 A g^−1^) [83].

Fiber-shape Na_3_V_2_(PO_4_)_2_F_3_@N-doped carbon (NVPF@C) can also be fabricated using the similar electrospinning method [77]. It is noteworthy that by replacing NVP with NVPF, the as-prepared NVPF@C nanowires show a higher output voltage of ~4.0 V (i.e., three steps of 4.0, 3.6, and 3.3 V) in the potential window of 2.0–4.3 V vs. Na^+^/Na (full cell with hard carbon anode showing three voltage plateaus of 3.9, 3.4, and 3.0 V between 2.2–4.3 V), and high rate capabilities (110, 102, 89, and 85 mA h g^−1^ at 0.1, 1, 10, and 20C, respectively) and long-term cycling stability (capacity retention of 87.8% after 1000 cycles at 20C and 83.4% retention at 50C after 1500 cycles) can be achieved. For full cells, they could deliver an average operating voltage of ca. 3.4 V and a promising energy density of 357 W h kg^−1^ (calculated on a reversible capacity of 105.1 mA h g^−1^ for the cathode; and an initial capacity of 73.2 mA h g^−1^ and an 89.9% retention after 150 cycles at 1C for the full cell could be obtained when configured with a hard carbon anode of ~150 mA h g^−1^).

**Figure 18:**
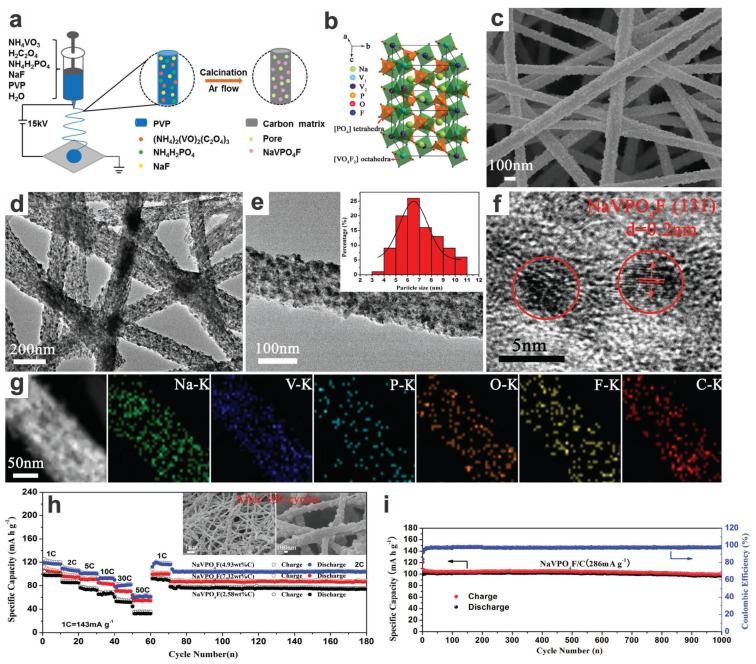
(**a**) Schematic illustration of the preparation of NaVPO_4_F/C nanofiber membrane electrode via electrospinning and calcination, and (**b**) the corresponding crystal structure of monoclinic NaVPO_4_F. (**c**) SEM image and (**d**–**f**) TEM images of NaVPO_4_F/C nanofibers with increasing magnification (inset: particle size analysis), as well as (**g**) the corresponding elemental mapping. Sodium storage performance of NaVPO_4_F/C nanofiber membrane as self-standing electrode of SIBs: (**h**) rate capabilities with different carbon contents (inset: SEM images of the NaVPO_4_F/C cathode after 180 cycles), and (**i**) long-term cycling performance at 2C (NaVPO_4_F/C with optimized 4.93 wt% C). Adapted with permission from Ref. [36]. Copyright 2017 WILEY-VCH Verlag GmbH and Co. KGaA, Weinheim.

Free- or self-standing electrodes are beneficial to the fabrication of flexible and high-energy-density energy storage devices due to the elimination of binders, metal current collectors, and additional conducting additives. Jiao group fabricated a NaVPO_4_F/C nanofiber membrane as free-standing cathode material via electrospinning (of a precursor solution of NH_4_VO_3_, NH_4_H_2_PO_4_, NaF, H_2_C_2_O_4_, and carbon source polyvinyl pyrrolidone (PVP)), followed by heat treatment (i.e., pyrolysis and annealing at 750 °C for 4 h in Ar atmosphere) (Figure 18a–c) [36]. The active NVPF nanoparticles with an average size of ~6 nm are embedded in the (meso)porous fibrous carbon matrix (Figure 18d–g). For sodium storage, it demonstrated a high discharge capacity of 120 mA h g^−1^ at 1C, an excellent rate capability of 61 mA h g^−1^ at 50C, as well as an ultralong cyclability with a capacity retention of 96.5% over 1000 cycles at 2C (Figure 18h,i). The superior long-term electrochemical stability and high rate performance can be attributed to the synergistic effect of the specific structures with nanosized NVPF and a 3D porous conductive network (4.9% carbon content), which maintains structural stability (restraining aggregation and detachment), enhances electrolyte permeation, and facilitates electron/ion transfer. It should be noted that an ideal long-potential platform at ca. 3.6/3.4 V (vs. Na^+^/Na) can be realized; however, a more appropriate anode should be chosen for a higher voltage output for promising applications, in which the hard carbon materials usually outperform those high-potential anode materials such as oxides and NASICON-type materials. Although dendrite issues may occur in alkali metal anodes with lower potential platforms, it is another topic that is gradually being solved by the delicate anode/electrolyte modification and design, i.e., suppressing the dendrite growth. Similar electrospun 3D flexible Na_3_V_2_(PO_4_)_3_/C (NVP/C) nanofiber membrane is also investigated [114], and the similar operating voltage and cycling performance are achieved comparable to those of the abovementioned NaVPO_4_F/C nanofiber membrane. For higher operating voltages of these flexible and free-standing cathodes, Na_3_(VO)_2_(PO_4_)_2_F nanorod arrays on carbon fibers are used as SIB cathodes via in situ conversion of VO_2_ nanosheet arrays on electrospun flexible carbon nanofibers in a hydrothermal autoclave [115]. The as-prepared flexible NVOPF cathode has two higher discharge plateaus of ca. 4.0 and 3.6 V as well as a specific capacity up to 117 mA h g^−1^ (at 100 mA g^−1^) and stable cycling performance with capacity degradation of 0.003% per cycle over 4500 cycles (i.e., 80 mA h g^−1^ at 500 mA g^−1^). And a high energy density of 221 W h kg^−1^ and power density of 9.4 kW kg^−1^ as well as high voltage output (average ~3.0 V) and long lifespan with 94.5% capacity retention at 1.0 A g^−1^ after 1200 cycles could be realized in a full cell with homologous flexible VO_2_ arrays as anodes (i.e., double vanadium-based flexible full cell). Some other layered vanadium oxide hydrates, such as H_2_V_3_O_8_ nanowire membrane, can also work as flexible additive-free cathodes. A high specific capacity of up to 168 mA h g^−1^ (around 1.8 Na^+^ ions insertion per unit formula) can be obtained with a sloping voltage plateau of ~3 V [116]; however, the rate capacities may be further improved with additional carbon coating, for example, to enhance the conductivity. Further annealing and Na-preintercalation/stabilizing will enhance the electrochemical stability and rate capabilities [117].

### 3.3. K-Ion Batteries

**Figure 19:**
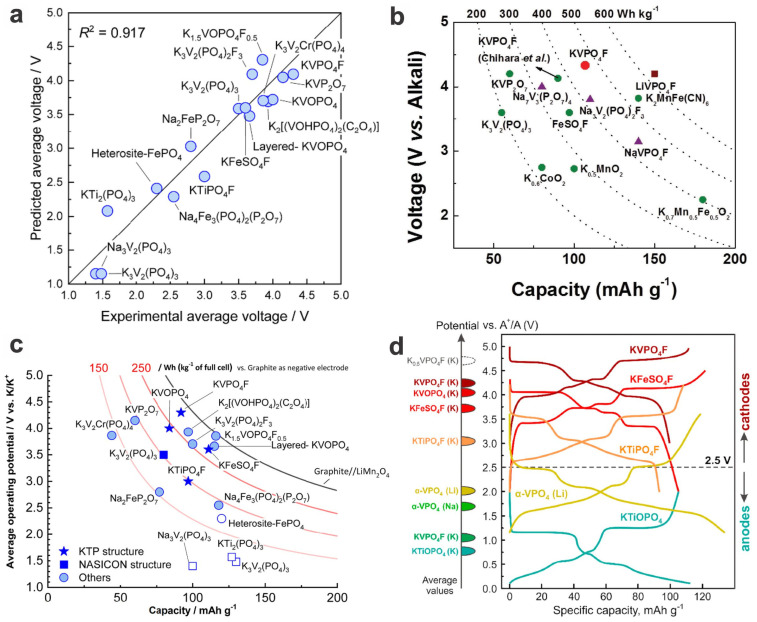
(**a**) Predicted vs. experimental average discharge voltage plot from the multiple linear regression analysis of selected polyanionic compounds for PIBs. (**b**) Capacity–voltage plots of selected cathode materials for PIBs (compared to typical LIBs and SIBs). (**c**) Average operating potential vs. specific capacity plot of selected polyanionic compounds for PIBs, as well as the energy densities of the K-ion full cells with graphite as anode. (**d**) Comparison of the average operating potentials and GCD curves of KTP-based and related electrode materials for PIBs. (**a**,**c**) Adapted with permission from Ref. [82] Copyright 2023 Author(s). Published by IOP Publishing Ltd., Open Access under the terms of the Creative Commons Attribution 4.0 license. (**b**) Adapted with permission from Ref. [120]. Copyright 2018 WILEY-VCH Verlag GmbH and Co. KGaA, Weinheim. (**d**) Adapted with permission from Ref. [121]. Copyright 2020 Elsevier B.V.

K-ion batteries (PIBs) are also one of the alternative secondary batteries to LIBs due to their sustainability and low cost, especially in large-scale energy storage (i.e., stationary or “grid” energy storage) [118]. For PIBs, the polyanionic phosphate-based compounds generally exhibit much better structural stability as well as an expanded voltage window over the layered transition-metal oxides, Prussian blue analogs, and organic crystals during repeated insertion and extraction, thus receiving considerable attention (for their comparison on the aspects of operating voltages, specific capacities, and energy densities, see Figure 19) [80,119].

**Figure 20:**
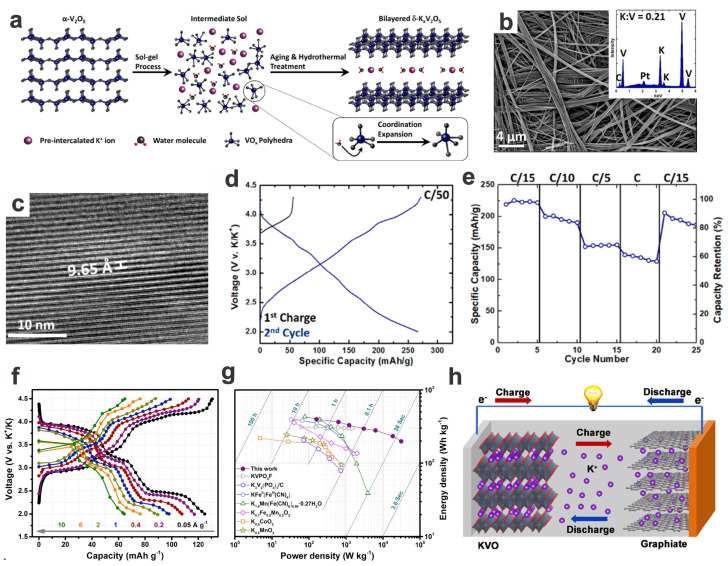
(**a**) Schematic illustration of a chemical preintercalation synthesis of bilayered *δ*-K*_x_*V_2_O_5_·*n*H_2_O nanobelts, in which K^+^ ions were incorporated in the interlayer space of bilayered V_2_O_5_. (**b**,**c**) SEM image (inset: EDX spectrum) and HRTEM image (interlayer spacing of 9.65 Å) of the well-formed nanobelts. Electrochemical properties of the as-synthesized *δ*-K_0.42_V_2_O_5_·*n*H_2_O nanobelts: (**d**) GCD curves at 0.02C, and (**e**) rate performance in the potential window of 4.3−2.0 V; (**f**) GCD curves of a single-crystalline bilayered *δ*-K_0.51_V_2_O_5_ (KVO) nanobelt electrode at various current densities; (**g**) Ragone plots of the KVO cathode compared with other advanced PIB cathodes; and (**h**) Schematic illustration of a K-ion full cell with KVO as cathode and graphite as anode. (**a**–**e**) Adapted with permission from Ref. [125]. Copyright 2018 American Chemical Society. (**f**–**h**) Adapted with permission from Ref. [127]. Copyright 2018 Elsevier Inc.

Some layered potassium vanadium oxide-based cathodes of PIBs have also been synthesized, e.g., layered K_0.5_V_2_O_5_ nanowires [122], K_0.83_V_2_O_5_ nanoparticles [123], although these usually exist in the form of hydrates (i.e., K_0.5_V_2_O_5_·0.5H_2_O [124], K*_x_*V_2_O_5_·*n*H_2_O [125], V_10_O_24_·*n*H_2_O [126]). These layered V-based oxides may have a high initial voltage of around 4 V despite no obvious voltage plateaus. Bilayered vanadium oxide (*δ*-V_2_O_5_) is a promising cathode candidate or precursor for alkali-metal-ion batteries, including non-aqueous PIBs, which is a low-cost alternative to LIBs. The Pomerantseva group fabricated a novel kind of K*_x_*V_2_O_5_·*n*H_2_O (KVO) nanobelts via chemical preintercalation of bilayered *δ*-V_2_O_5_ (Figure 20a,b). As a cathode for PIBs, the as-prepared 1D *δ*-K_0.42_V_2_O_5_·0.25H_2_O nanobelt with an expanded interlayer distance of 9.65 Å can exhibit high initial discharge capacities (268 and 226 mA h g^−1^ at 0.02 and 0.067C, respectively, in the potential window of 4.3–2.0 V vs. K^+^/K), as well as good rate capacities (58% capacity retention at 1C) and fair cycling performance (74% capacity retention after 50 cycles at 0.067C) (Figure 20c–e). Although the discharging curve shows a sloping feature resembling that of capacitors, mechanism analysis reveals a diffusion-controlled intercalation dominating over surface-based nonfaradaic capacitive contribution (viz., mostly pseudocapacitive contribution rather than double-layer capacitance), and therefore contributing to the high electrochemical performance [125]. And via delicately optimizing the growth orientation and structures thereof, the further synthesized single-crystalline bilayered *δ*-K_0.51_V_2_O_5_ nanobelts could exhibit distinct three voltage plateaus (at around 3.9, 3.2, and 2.5 vs. K^+^/K) with an average voltage of 3.2 V, a relatively high capacity of 131 mA h g^−1^ (corresponding to approximately 1 K^+^ insertion per formula), and significantly enhanced rate capabilities (e.g., 97 and 64 mA h g^−1^ at 1 and 10 A g^−1^, respectively) (Figure 20f). The enhanced rate capabilities should be attributed to the unique 1D morphology with an inner large interlayer spacing structure (viz., K^+^ diffusion in the ample interlayer space and basically perpendicular to the nanobelt growth direction, leading to the lowest diffusion barrier and shortest diffusion distance). The as-prepared 1D KVOs as cathodes showed high energy and power densities (superior to most other typical PIB cathode materials), not only in the half-cell configuration (energy/power densities of 397 W h kg^−1^ and 30.8 kW kg^−1^) but also in the full cells assembled with classic graphite anodes (e.g., average operating voltage of 2.5 V, energy/power densities of 238 W h kg^−1^ and 5480 W kg^−1^ for the full cell) (Figure 20g,h). Through ex situ XRD and XPS analyses, a biphasic transition happens during the K^+^ insertion and extraction (i.e., stage II with a new phase while the other two stages are both in the single phase, all with a stable bilayered structure for K^+^ accommodation), and the V^4+^/V^5+^ redox couple contributes to the charge–discharge voltage plateaus [127]. It is interesting that the nanocrystalline K_0.5_V_2_O_5_·0.5H_2_O (with reduced long-range structural order and suppressed phase transition) synthesized by the sol–gel method shows a sloped discharge profile, while the highly crystalline K_0.5_V_2_O_5_ by hydrothermal reaction shows initially multiple discharge plateaus and afterwards sloped curves in the voltage window of 4.5–2.0 V, although the nanocrystalline KVO possesses higher rate capabilities and cyclability [124]. These works may pave the way for low-cost, room-temperature PIBs for sustainable large-scale energy storage.

High-voltage PIBs are more desirable, and polyanionic vanadium fluorophosphates are promising candidates. One of the typical vanadium-based cathodes is polyanionic K_3_V_2_(PO_4_)_2_F_3_ (K_3_VPF), which can be derived from the Na_3_V_2_(PO_4_)_2_F_3_ analogs abovementioned. The KVPF-based cathode materials for PIBs can tolerate large-size K^+^ ions compared to Li^+^ ions but are showing a small volume change (i.e., 6.2%) during K^+^ ion intercalation/deintercalation. What is most noteworthy is the high average potential of the cathode, ~3.7 V vs. K^+^/K, arising from the binary plateaus (i.e., ca. 4.3 V and 3.4 V), similar to its Na-based analogs; when configured with the graphite anode, a 3.4 V output could be realized. The high energy density of 385 W h kg^−1^ and the high voltage output render it a great opportunity in large-scale energy storage as a promising competitor to SIBs. The Zhang group prepared the K_3_V_2_(PO_4_)_2_F_3_ powder by electrochemical ion exchange of its Na-based analog in a KPF_6_ organic electrolyte [80]. However, during the repeated cycling, the full PIB shows a gradual decay both in capacity and voltage output, i.e., the capacity decreasing from over 80 mA h g^−1^ to around 60 mA h g^−1^, and the voltage dropping by about 0.2 V, although its stabilized cycling comes after the initial 20 cycles. It is showing superior performances than its K_3_V_2_(PO_4_)_3_-based counterparts, such as the 3D conductive network K_3_V_2_(PO_4_)_3_/C nanocomposites [128], not only in terms of high-voltage plateaus but also in terms of specific capacities and rate capabilities. 

**Figure 21:**
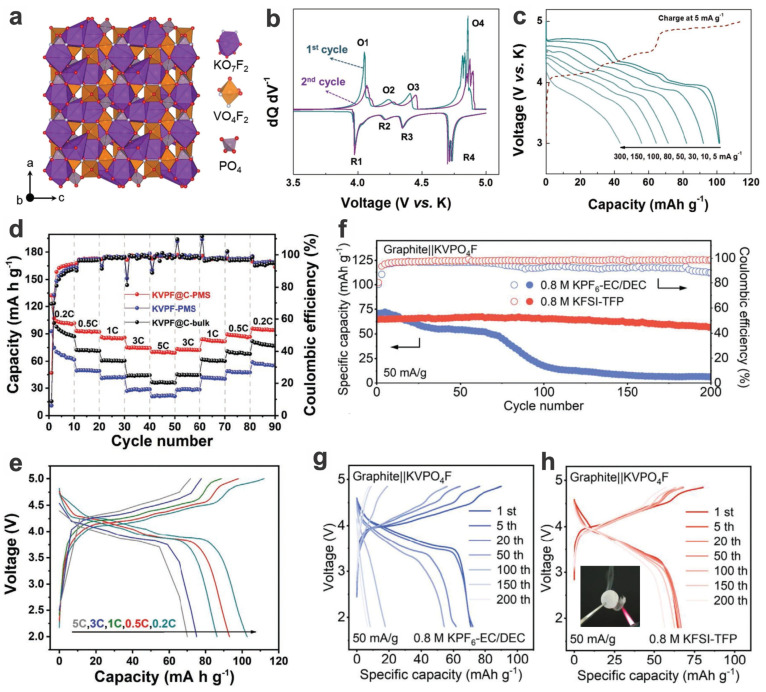
(**a**) Crystal structure of the high-voltage cathode material KVPO_4_F projected along *b*-axis. (**b**) Differential capacity curves and (**c**) rate capability of KVPO_4_F as cathode material for PIBs. Adapted with permission from Ref. [120]. 2018 WILEY-VCH Verlag GmbH and Co. KGaA, Weinheim. (**d**) Rate capabilities of various KVPF samples (PMS with and without carbon coating, bulk one). (**e**) GCD curves of KVPF@C-PMS at different rates in the potential window of 5.0–2.0 V. Abbr. KVPF@C-PMS: pomegranate-like micro/nano hierarchical carbon-coated KVPO_4_F microspheres. Adapted with permission from Ref. [129]. Copyright 2022 Wiley-VCH GmbH. (**f**) Cycling performances and (**g**,**h**) GCD profiles of KVPO_4_F||graphite full cells with different electrolytes, i.e., 0.8 m KPF_6_–EC/DEC and 0.8 m KFSI–TFP at 50 mA g^−1^ in the potential window of 4.85–1.8 V (inset: flammability test of KFSI–TFP electrolyte). Adapted with permission from Ref. [131]. Copyright 2023 Wiley-VCH GmbH.

KVPO_4_F cathode material (in the form of nanoparticles prepared by stoichiometric sintering, KVPF) can demonstrate a reversible capacity of ~105 mA h g^−1^ at a mean voltage of ~4.3 V (vs. K^+^/K) and a high gravimetric energy density of ~450 W h kg^−1^ (Figure 21a–c). Various intermediate phases upon K^+^ insertion/extraction are revealed via ex situ XRD analysis and ab initio calculations, and partial O_2_^−^ substitution of F^−^ (or called oxygenation) in KVPO_4_F leads to anion-disordered structure (along with partial oxidation of V^3+^ and reduced inductive effect) and therefore a smoother and sloped voltage feature with no electrochemical plateaus due to the disruption of K^+^/vacancy ordering, although the reversible capacity and average voltage decrease with the lowering fluorine content. However, the improvement in rate capabilities and cycling performances of these KVPF materials is needed for further competitive applications [120]. Through further carbon coating/compositing and the preparation of micro-sized aggregated particles, the overall performance may be enhanced. Xie et al. fabricated a kind of pomegranate-structured carbon-coated KVPF microspheres comprised of nanosized primary particles and carbon sheets, which simultaneously improve the K^+^ diffusion kinetics and cycling stability. Also the dense hierarchical structure endows it with a higher compact density of 2.45 g cm^−3^ and a high volumetric energy density of up to 891 W h L^−1^, along with an excellent capacity retention of 85% over 200 cycles, a high capacity of 102 mA h g^−1^ at 0.3C and significantly enhanced rate capabilities (Figure 21d,e) [129]. And the performances, including rate capabilities, ICE, and cycling performance, could be further enhanced by in situ carbon coating (e.g., the porous single-crystalline KVPO_4_F/C nanoplates) [130]. Electrolytes also have an impact on the performance of these alkali-metal batteries, e.g., 1 m KPF_6_ EC/PC (1:1 *v*/*v*) endows them with higher specific capacities and average output voltage than 0.6 m KPF_6_ PC/FEC (EC: ethylene carbonate, PC: propylene carbonate, PC: fluoroethylene carbonate) [129]. Although FEC can be added to the electrolytes to alleviate overcharge behavior and electrolyte decomposition, it may weaken the performance of PIBs in specific capacities and voltage output. A specific fluorinated phosphate solvent, i.e., tris(2,2,2-trifluoroethyl) phosphate (TFP) (in the classic KFSI system, KFSI: potassium bis(fluorosulfonyl)imide), may serve as a promising electrolyte for high-voltage PIBs with KVPF cathodes. The high oxidative stability and weak solvating ability enable ultrahigh-voltage stability up to 4.95 V and high capacity detention (e.g., 88% over 200 cycles for cathodes and 87% for full cells) while exhibiting extraordinary compatibility with high-voltage cathodes, graphite anode, and Al-based current collector. Moreover, it also shows a favorable safety profile for practical applications due to the enhanced thermal stability and non-flammability (Figure 21f–h) [131]. 

Furthermore, a new kind of layered KVOPO_4_ (KVOP), e.g., in the form of nanosheet, shows slightly lower operating voltages (average discharge voltage of 3.65 V), high reversible capacity of 115 mA h g^−1^ at 0.2C in the potential window of 4.6–2.0 V (vs. K^+^/K), high coulombic efficiency (98.5%), excellent rate capacities (107, 98, 92, and 83 mA h g^−1^ at 1, 5, 10, and 20C, respectively), and superior long-term cycling stability (capacity retention of 86.8% after 100 cycles at 0.2C and 75.6% after 500 cycles at 5C), demonstrating better performances as cathode material for PIBs than its microflower-like and bulk counterparts, not only in rate capabilities but also in cycling stability [132]. Some layered mixed-polyanions, e.g., K_2_[(VOHPO_4_)_2_(C_2_O_4_)] (KVPox) [133], are showing similar structures and performances as those of layered KVOP. The specific fluorinated electrolyte, i.e., KFSA/DME [potassium bis(fluorosulfonyl)amide in dimethoxyethane], endows it with higher specific capacity and coulombic efficiency than traditional KPF_6_/carbonate ester electrolytes. Owing to the facile K^+^ ion migration in the open framework, the as-synthesized KVPox/reduced graphene oxide (G-KVPox) composite material could demonstrate a high output voltage of 4 V (average voltage of 3.85 V), an outstanding rate capability (80 mA h g^−1^ at 10C), as well as good long-term cycling stability (capacity retention of 67% after 500 cycles at 2C) [134]. In addition, pea-shaped KVP_2_O_7_@C particles synthesized by spray drying and annealing could show a reversible capacity of 60 mA h g^−1^ at 0.2C with two distinct discharge plateaus (4.5 and 4.1 V vs. K^+^/K), accounting for a high energy density of ca. 250 W h kg^−1^. When configured with a hard carbon anode, the full cell exhibited a high average operating voltage of ~3.6 V, a high energy density of 184 W h kg^−1^, as well as superior cycling stability (i.e., 89% capacity retention over 400 cycles). More information on these vanadium-based low-dimensional high-voltage cathode materials for PIBs is listed in Table 2, compared with those for LIBs and SIBs.

**Table 2:** Summary of the performances of typical vanadium-based high-voltage cathode materials and some of their full cells thereof.

Types of Materials	Methods	Current Density	Cycle No.	Cut-Off Voltage [V]	SC [mA h g^−1^]	CR (%)	CE [%]	ICE [%]	Initial Discharge/Charge Capacities [mA h g^−1^]	Rate Capacity (Current Density) [mA h g^−1^]	Year/Ref.
LIBs cathodes											
Li_3_V_2_(PO_4_)_3_/C nanobelts (aggregates)	Ball milling, solid-state reaction	1C	160	3.0–4.3	130	99.2	~100	99.2	131/132	131, 128, 122, 110 (1, 2, 4, 8C)	2011 [40]
Li_9_V_3_(P_2_O_7_)_3_(PO_4_)_2_ nanotubes	Molten salt	0.5C	300	2.5–4.6	147	95.0	~97	92.4	155/167	143, 131, 123, 104, 81, 63 (1, 2, 5, 10, 20, 40C)	2015 [75]
(NH_4_)_2_V_7_O_16_ particles	Hydrothermal, ball milling	0.02 A g^−1^	50	1.3–3.2	181	~84	>99	94.5	219/232 (0.01 A g^−1^)	215 (0.01) 103 (0.2)	2020 [32]
		0.02 A g^−1^	20	1.5–3.0	61	~87	>99	73.9	71.4/96.6	70 (0.02)	
SIBs cathodes											
*γ*-Na_0.96_V_2_O_5_ particles	Soft chemistry synthesis (sodiation)	0.2C	50	1.75–4.0	112	~90	~100	~100	125/125	120, 85 (0.2, 2C)	2018 [94]
Na_3_V_2_(PO_4_)_3_@C core-shell nanoparticles (~40 nm)	Hydrothermal (surfactant-assisted), calcination	5C	700	2.5–3.8	91	96.1	~100	93	104/112	104, 95 (0.5, 5C)	2014 [37]
Na_3_V_2_(PO_4_)_3_ nanofiber	Hydrothermal, annealing	10C	1000	2.3–3.9	96	95.9	~100	–	–	113, 110, 108, 98, 94 (1, 5, 10, 50, 100C)	2016 [38]
Porous 1D Na_3_V_2_(PO_4_)_3_@C nanowires	Hydrothermal (surfactant-assisted), freeze-drying, post-heating	1C 20C	1000	2.3–3.9	97 74	93 81	~100	98	107/109	104, 104, 99, 96, 84, 77, 62) (0.5, 1,10, 20, 40, 50, 60C)	2016 [8]
Mo-doped Na_3_V_2_(PO_4_)_3_@C nanowires	Electrospinning, annealing	1C 5C	2000	2.5–4.0	115 101	95.3 93.0	–	~97	117/120 (0.1C)	99, 93, 80 (50, 100, 150C)	2021 [113]
	Full cell (with hard carbon, HC, as anode) ^b^	5C	8000	2.3–3.8	87	85.7	–	94.5	111/117	109, 102, 98, 90, 79 (0.5, 10, 30, 50, 100C)	
3D Na_3_V_2_(PO_4_)_3_ @C@rGO	Freeze-drying, calcination (Ar-5%H_2_)	100C	10,000	2.5–3.8	55	64	~100	~98	~117/~120 (0.5C)	115, 112, 103, 91, 86 (1, 30, 50, 80, 100C)	2015 [45]
Layered NaVOPO_4_/C composite	Refluxing, hydrothermal, ball-milling	0.5C	1000	2.0–4.2	75	67	>99.5	~95	144/152 (0.02)	144, 130, 118, 112, 96, 80, 58 (0.05, 0.1, 0.2, 0.5, 1, 2, 5C)	2018 [105]
Ultrafine Na_7_V_3_(P_2_O_7_)_4_^b^	Sol–gel, calcination	2C 10C	100	2.5–4.2	~55 ~33	~97 92	–	~73	73/~100	~72/66/36 (0.025, 1, 10C)	2016 [54]
Na_7_V_4_(P_2_O_7_)_4_PO_4_ nanorods	Sol–gel, calcination	0.05C 0.5C	200	2.5–4.1	88 82	95.2 92.6	–	~94	92/~98 (0.05C)	73 (10C)	2014 [56]
Fiber-shape Na_3_V_2_(PO_4_)_2_F_3_ @N-doped carbon	Electrospinning, calcination	5C 20C 50C	200 1000 1500	2.0–4.3	94 72 60	97.1 87.8 83.4	–	78.3	102/130 (5C)	110, 102, 89, 85, 79 (0.1, 1, 10, 20, 30C)	2020 [77]
	NVPF|| HC ^b^	1C	150	2.2–4.3	82	82.0	~100	<80	–	100 (1C)	
Na_3_V_2_(PO_4_)_2_F_3_@C nanocomposite	Sol–gel, sintering	10C 30C	1000 3000	2.0–4.3	~37 68	70 50	>97	91	–	133,129, 112, 85, 74, 57 (0.5, 1, 5, 10, 15, 30C)	2015 [78]
Carbon-coated Na_3_(VOPO_4_)_2_F nanocomposite	Mechanochemical synthesis	20C	10,000	2.5–4.2	111	98	~100	~90	112/125	142, 113 (0.1, 20C)	2021 [43]
	NVOPF/KB ||HC ^b^	2C	100	2.0–4.2	61	90.7	~100	~76	94/123	94, 87, 74, 67, 58, 49 (0.2, 0.5, 1, 2, 5, 10C)	
Na_3_V_2_(PO_4_)_1.95_ (SiO_4_)_0.05_O_2_F nanocubes	Hydrothermal, annealing	0.5C 10C	700 1000	2.0–4.3	121 114	95.6 ~100	~100	98	126/128 (0.5C)	126, 126, 123, 118, 110, 93, 76 (0.5, 1, 2, 5, 10, 20, 30C)	2023 [29]
	Full cell (HC, pre- sodiated)	1C 5C	100 300	2.0–4.3	122 108	97.7 92.3	99.8	92.4	126/136	126, 91 (0.2, 15C)	
RuO_2_-coated Na_3_V_2_O_2_(PO_4_)_2_F nanowires	Hydrothermal (microemulsion-mediated), hydrolysis precipitation	20C	1000	2.5–4.3	95	90.5	99.3	94.0	126/134 (0.1C)	120 (1C) 110 (20C) 70 (40C)	2015 [19]
NaVPO_4_F/C nanofiber membrane	Electrospinning, carbonization	2C	1000	2.6–4.5	~99	96.5	~100	~95	~120/126	120, 111, 105, 101, 82, 61 (1, 2, 5, 10, 30, 50C)	2017 [36]
PIBs cathodes											
K_3_V_2_(PO_4_)_2_F_3_ powder	Solid-state reaction, electrochemical ion exchange	10 20 (mA g^−1^)	100 180	2.0–4.6	101 90	97 95	–	~74	104/140	104, 83, 50 (0.1, 1, 2.5C)	2019 [80]
	KVPF||graphite ^b^	–	50	1.5–4.5	59	70.2	>96	67	84/126	–	
Bilayered *δ*-K_0.51_V_2_O_5_ nanobelts	Chemical preintercalation, hydrothermal	0.1 2 (A g^−1^)	100	2.0–4.5	78 51	61.3 60.5	~100	–	131/69 (0.03 A g^−1^)	125, 117, 97, 89, 64 (0.05, 0.2, 1, 2, 10 A g^−1^)	2019 [127]
	KVO||graphite ^b^	0.3 (A g^−1^)	100	1.8–3.9	67	84	~91	–	94/44 (0.1 A g^−1^)	94, 60 (0.1, 2 A g^−1^)	
Layered KVOPO_4_ nanosheets	Hydrothermal, chemical potassiation	0.5C 5C	100 500	2.0–4.6	~98 63	86.8 75.6	98.5	~91	115/126 (0.2C)	113, 107, 103, 98, 92, 83(0.5, 1, 2, 5, 10, 20C)	2019 [132]
KVPO_4_F/C nanoplates	Hydrothermal, annealing	0.5C 10C	100 1000	2.0–5.0	93 80	92.4 82.5	~99	~86	135/116 (0.2C)	107, 100, 99, 93, 74 (0.2, 5, 10, 50, 100C)	2022 [130]
	KVPO_4_F/C||soft carbon	0.5C	500	1.5–4.8	76	80.3	~99	~52	180/93	95, 89, 87, 69 (0.5, 5, 10, 20C)	
Pomegranate-like carbon-coated KVPO_4_F microspheres ^a^	Hydrothermal, calcination	0.3C	100	2.0–5.0	93	91.4	~95	~42	272/114	103, 93, 86, 70 (0.2, 0.5, 1, 5C)	2022 [129]
K_2_[(VOHPO_4_)_2_ (C_2_O_4_)]/rGO composite (particles)	Precipitation, dehydration	2C 0.1C	150 /500 100	2.5–4.5	85/60 94	95/67 96	97.4	87.5	98/112 (0.1C)	100, 98, 95, 92, 84, 80 (0.2, 0.5, 1, 2, 5, 10C)	2019 [134]
Pea-shaped KVP_2_O_7_@C particles	Spray drying	0.5C	100	2.0–4.95	45	88	~95	~59	60/102	60, 52, 46, 36 (0.2, 0.5, 1, 3C)	2021 [135]
	KVP||graphite ^b^	0.5C	400	1.0–4.9	46	89	99	~90	52/58	52 (0.5C)	

Notes: Abbr. SC: specific capacity; CR: capacity retention; CE: coulombic efficiency; ICE: initial coulombic efficacy. ^a^ Primary nanoparticles aggregate into microsized secondary particles. ^b^ Performances of the cathode in a full cell configured with hard carbon (HC) or graphite as anode.

## 4. Conclusions and Perspectives

Vanadium-based cathode materials with specific low-dimensional features and intrinsic advantages will provide huge possibilities in future electrochemical energy storage systems. The vanadium phosphates, fluorophosphates, oxides, and vanadates will all have their own specific and promising applications in next-generation metal-ion batteries, not only in Li-ion batteries but also in post-Li-ion batteries, including Na-ion and K-ion batteries. The metal anode batteries are also promising due to the recent significant progress on electrolytes and anode design/modification. The insights and possible future development directions are summarized as follows:

1. Polyanionic vanadium phosphates and fluorophosphates and their analogs/derivatives have higher redox potential, structural/thermal stability, operational safety, and cycle life compared to vanadium oxides or vanadates; however, the latter ones usually show higher capacity. Some vanadium oxide bronzes have attracted significant attention as cathode materials for alkali-metal or alkali-metal-ion batteries due to their layered and open-framework structures, as well as their superior thermal stability, moisture tolerance, and high electrochemical reactivity. They may serve as high-voltage cathode materials, although their cycling performance needs further improvement to compete with these polyanions, such as vanadium phosphates. Thus, their utilization needs a balanced and comprehensive evaluation; more parameters, including cost, yield, and productivity, should be considered for practical application scenarios.
2. Solid state reactions are a facile way to synthesize these vanadium-based materials, especially for vanadium polyanionic compounds, of which stoichiometric amounts of precursors such as alkali-containing salts (Li, Na, or K-containing), vanadium-containing compounds (e.g., V_2_O_5_, sodium metavanadate, vanadium acetylacetonate), phosphates, and/or carbon sources (reductant/conductor), NaF are usually needed, ball-milling, hydrothermal/solvothermal methods, sol–gel methods, followed by annealing/calcination under N_2_, Ar, or Ar/H_2_ atmosphere are usually needed. Although the hydrothermal/solvothermal methods can be directly used for the synthesis of these vanadium-based materials with ideal 0D, 1D, 2D, or even 3D (with the assistance of freeze drying), their harsh conditions (i.e., high pressure and high temperature of liquids) may not be suitable for low-cost, large-scale industrial production. Thus, the (high-energy) ball-milling or sol–gel methods followed by further annealing/calcination will be the more efficient strategies for ultimate mass production.
3. Structure regulation and crystallinity are of great importance to guarantee the vanadium-based materials optimal performances. The electrode and full cell engineering also affect the performances of the device; e.g., 3D open and highly conductive frameworks of the electrode generally show a higher utilization ratio of active materials as well as enhanced ionic/electronic transport and diffusion, thus resulting in higher rate capacities and higher power densities thereof. In addition, the cost-effectiveness, the phase and structure stability are also of great importance for practical application, e.g., although with high capacity, some materials do not have a stable phase in air and some may undergo structural damage during electrochemical deep cycling. The carbon-coating, nanostructuring, ion-doping, and compositing/hybridization are typical methods to enhance the conductivity for high performances.
4. The high-plateau capacity ratio plays a key role in effective application since it will demonstrate enhanced energy density and a decreased number of batteries connected in series. And the mechanism of large voltage drops or significant polarization should be further studied to optimize the full cell performance. In operando, XRD analysis and density-functional theory (DFT) calculations are efficient ways to give fascinating insights into the joint optimization of structures and performances.
5. Overcharge behavior and significant electrolyte decomposition may happen along with these high-voltage cathodes. Proper electrolytes that can effectively alleviate overcharge behavior (for high initial coulombic efficiency, ICE) and not weaken the electrochemical performances (e.g., specific capacity, voltage polarization) are urgently needed for practical application. For high-voltage cells (e.g., up to 4.8 V), electrolyte additives such as fluoroethylene carbonate (FEC) and tris(2,2,2-trifluoroethyl) phosphate (TFP) may be added for stabilization. New electrolytes with higher electrochemical stability and cyclability, such as ionic liquids, may be adopted.
6. The good match with appropriate anode materials is of great importance, e.g., these vanadium-based materials matching with highly conductive hard carbon will endow the full cell with high voltage output, especially for SIBs. However, the full cells of SIB now often encounter low rate performance, viz., the rate performance, powder densities, and voltage output need further improvements, and the modification, optimization, and matching of hard carbons are critical for competitive applications. The ICE of the hard carbon-based anodes should also be evaluated and enhanced for practical application. Pre-lithiation, pre-sodiation, or pre-potassiation is usually adopted to form stable solid-electrolyte interphase (SEI) layers on the hard carbon anode for higher ICE by reducing the irreversible capacity loss for the full cells. The compatibility of the anode materials with the cathode materials is also vital. In addition, the classic hard carbon materials, some vanadium oxide bronze-based anode materials are showing high capacity and a high average discharge plateau for the full battery; however, they may decay fast when poor compatibility exists.
7. Flexible and freestanding electrodes are promising for flexible, wearable batteries and smart devices. Vanadium-based materials are playing a distinct role in the fabrication of these high-performance cathodes as well as anodes, although frontier research is still in its infant stage. More effort should be put into the enhancement of mechanical strength, flexibility, battery design, energy/power densities, cycling performances, and safety.
8. Although some nanostructured V-based cathode materials are showing excellent performance in a coin-cell configuration with low massive loading (ca. 1–2 mg cm^−2^), this does not necessarily guarantee their large-scale implementation in commercial batteries. Due to high mass loading and sufficiently high tap density, as well as enough adhesion to the Al current collector, nanostructured materials usually have a high surface area but low tap density (or compact density, an important parameter in the production of alkali-ion batteries), thus possibly stripping them from the current collector and making them unsuitable for further assembly. So structural regulation is needed, and moderate aggregation is beneficial to higher performance in practical applications of electrochemical energy storage and conversion.

## 5. Patents

1. W. Ni, Preparation method of high-purity vanadium pentoxide nanofiber non-woven fabric (CN113481656B), 2021. https://patents.google.com/patent/CN113481656B/en (accessed on 20 January 2024).
2. W. Ni, Low-cost room-temperature rapid batch preparation method and equipment for special-shaped vanadium oxide nanofibers and aggregates thereof (CN114293321B), 2021. https://patents.google.com/patent/CN114293321B/en (accessed on 20 January 2024).
3. W. Ni, Preparation method of porous nano vanadium oxide, porous nano vanadium oxide and application (CN116119713A), 2022. https://patents.google.com/patent/CN116119713A/en (accessed on 20 January 2024).
4. W. Ni, Hydrated sodium polyvanadate, sodium vanadium oxide nanofiber and aggregate, and preparation method (CN117187987A), 2023. https://patents.google.com/patent/CN117187987A/en (accessed on 20 January 2024).

## Figures and Tables

**Table 1:** Summary of the parameters of typical vanadium-based high-voltage cathode materials.

Cathode Materials	Structure Type (Space Group)	Potential Window (V)	Discharge Plateaus (V)	Theoretical Capacities (mA h g^−1^)	Experimental Capacities (mA h g^−1^)	Theor./Exp. Energy Densities (W h kg^−1^)	Year/Ref.
LIBs cathodes							
*γ*-LiV_2_O_5_	2D layered	4.0–2.0	3.5, 2.5	284	210 @0.02 A g^−1^	–	2022/[11]
*α*-Li_3_V_2_(PO_4_)_3_	NASICON Monoclinic (*P*2_1_/*n*)	4.4–3.0	4.1, 3.7, 3.6 (av. ~3.8 V)	197 (4.8–3.0 V) 133 (4.3–3.0 V) ^a^	131	–	2018/[13]
LiVPO_4_F	$\mathrm{Triclinic}\;(P\bar{1}$)	4.6–2.5	4.2	156	141@0.12C	655/592	2018/[26]
Li_9_V_3_(P_2_O_7_)_3_(PO_4_)_2_	Layered Trigonal $(P\bar{3}$*c*1)	4.6–2.5	4.5, 3.8	173.5	155@0.5C	–	2015/[75]
SIBs cathodes							
Na_3_V_2_(PO_4_)_3_	NASICON Rhombohedral $(R\bar{3}$*c*)	3.8–2.5	3.4	118	115@1C	401(394)/384	2015/[45]
NaVOPO_4_	Layered Monoclinic (*P*2_1_/*c*)	4.5–2.0	4.0, 3.6	145	130@0.1C	591 ^b^/~450	2014/[57] 2021/[76]
Na_3_V_2_(PO_4_)_2_F_3_	NASICON Tetragonal (*P*4_2_/*mnm*)	4.3–2.0	4.0/4.1 ^c^, 3.6, 3.3 (av. ~3.9/3.8 V)	128	110/102 @0.1/1C 130@0.5C	507(495) ^c^ /500	2020/[77] 2015/[78]
NaVPO_4_F	NASICON monoclinic (*C*2/*c*)	4.5–2.6	3.4	143	120@1C	485/–	2017/[36]
	Tetragonal (*I_4_*/*mmm*)	4.3–1.5	4.2, 3.7 (av. 3.95 V)	143	121/69 @0.05/0.5C	527/478	2015/[79]
Na_3_V_2_O_2_(PO_4_)_2_F ^d^	Pseudolayered Tetragonal (*I_4_*/*mmm*)	4.3–2.5	4.0, 3.6 (av. 3.8 V)	130	120 (1C)	494/479	2015/[19]
*β*-NaVP_2_O_7_	Monoclinic (*P*2_1_/*c*)	4.4–2.0	4.1, 3.8 (av. 3.9 V)	108	104, 96 (0.1, 1C)	421 ^e^/393	2019/[48]
Na_7_V_3_(P_2_O_7_)_4_	Quasi 2D Monoclinic (*C*2/*c*)	4.35–2.5	4.1	79.6	~80	329/–	2016/[53,54]
Na_7_V_4_(P_2_O_7_)_4_PO_4_	$\mathrm{Tetragonal}\;(P\bar{4}$2_1_*c*)	4.2–2.0	3.88	92.8	91@0.5C	357/–	2014/[55]
PIBs cathodes							
K_3_V_2_(PO_4_)_2_F_3_	NASICON orthorhombic (*Cmcm*)	4.6–2.0	4.3, 3.4 (av. ~3.7 V)	115	104/83 @0.1/1C	–/385	2019/[80]

Notes: Abbr. Theor./Exp.: Theoretical/Experimental. ^a^ Some data vary slightly from different sources [40,76]. ^b^ Theoretical energy density of the cathode calculated based on 3.8 V × 155.6 mA h g^−1^ = 591.3 W h kg^−1^, assuming 1.2 electrons are supplied (i.e., 155.6 mA h g^−1^) from the V^3.8+^/V^5+^ redox couple. ^c^ The first discharge potential may increase by ~0.1 V via lowering the discharge rate or scan speed in some systems [78]. ^d^ Or named Na_3_V_2_(PO_4_)_2_O_2_F, Na_3_(VO)_2_(PO_4_)_2_F, or Na_3_(VOPO_4_)_2_F. ^e^ Theoretical energy density of the cathode, calculated based on 108 mA h g^−1^ × 3.9 V.

## Data Availability

No new data were created or analyzed in this study. Data sharing does not apply to this article.

## References

[1] Xu S., Yang Y., Tang F., Yao Y., Lv X., Liu L., Xu C., Feng Y., Rui X., Yu Y. (2023). Vanadium fluorophosphates: Advanced cathode materials for next-generation secondary batteries. Mater. Horiz..

[2] Zhang S., Tan H., Rui X., Yu Y. (2020). Vanadium-based materials: Next generation electrodes powering the battery revolution?. Acc. Chem. Res..

[3] Xu X., Xiong F., Meng J., Wang X., Niu C., An Q., Mai L. (2020). Vanadium-based nanomaterials: A promising family for emerging metal-ion batteries. Adv. Funct. Mater..

[4] Ni W., Shi L.-Y., Gupta R.K. (2023). Rich structural chemistry of metal phosphates/phosphonates for emerging applications: V, Ti-containing materials. Metal Phosphates and Phosphonates: Fundamental to Advanced Emerging Applications.

[5] Bianchini M., Xiao P., Wang Y., Ceder G. (2017). Additional sodium insertion into polyanionic cathodes for higher-energy Na-ion batteries. Adv. Energy Mater..

[6] Chen G., Huang Q., Wu T., Lu L. (2020). Polyanion sodium vanadium phosphate for next generation of sodium-ion batteries—A review. Adv. Funct. Mater..

[7] Sharma L., Adiga S.P., Alshareef H.N., Barpanda P. (2020). Fluorophosphates: Next generation cathode materials for rechargeable batteries. Adv. Energy Mater..

[8] Jiang Y., Yao Y., Shi J., Zeng L., Gu L., Yu Y. (2016). One-dimensional Na_3_V_2_(PO_4_)_3_/C nanowires as cathode materials for long-life and high rate Na-ion batteries. ChemNanoMat.

[9] Cheng Y., Xia Y., Chen Y., Liu Q., Ge T., Xu L., Mai L. (2019). Vanadium-based nanowires for sodium-ion batteries. Nanotechnology.

[10] Balogun M.-S., Luo Y., Lyu F., Wang F., Yang H., Li H., Liang C., Huang M., Huang Y., Tong Y. (2016). Carbon quantum dot surface-engineered VO_2_ interwoven nanowires: A flexible cathode material for lithium and sodium ion batteries. ACS Appl. Mater. Interfaces.

[11] Ma J., Chen Y., Zhang Y., Song T., Wang X., Wu X., Law M.-K., Long B. (2022). In situ K doped γ-LiV_2_O_5_ as long-life anode and cathode for lithium ion battery. ACS Appl. Energy Mater..

[12] Kovrugin V.M., Chotard J.-N., Fauth F., Masquelier C. (2020). Na_7_V_3_(P_2_O_7_)_4_ as a high voltage electrode material for Na-ion batteries: Crystal structure and mechanism of Na^+^ extraction/insertion by operando X-ray diffraction. J. Mater. Chem. A.

[13] Sørensen D.R., Mathiesen J.K., Ravnsbæk D.B. (2018). Dynamic charge-discharge phase transitions in Li_3_V_2_(PO_4_)_3_ cathodes. J. Power Sources.

[14] Guo J.Z., Wang P.F., Wu X.L., Zhang X.H., Yan Q., Chen H., Zhang J.P., Guo Y.G. (2017). High-energy/power and low-temperature cathode for sodium-ion batteries: In situ XRD study and superior full-cell performance. Adv. Mater..

[15] Su B., Wu S., Liang H., Zhou W., Liu J., Goonetilleke D., Sharma N., Sit P.H.L., Zhang W., Yu D.Y.W. (2020). High-performance NaVO_3_ with mixed cationic and anionic redox reactions for Na-ion battery applications. Chem. Mater..

[16] Sijuade A.A., Eze V.O., Arnett N.Y., Okoli O.I. (2023). Vanadium MXenes materials for next-generation energy storage devices. Nanotechnology.

[17] Qin H., Liang S., Chen L., Li Y., Luo Z., Chen S. (2020). Recent advances in vanadium-based nanomaterials and their composites for supercapacitors. Sustain. Energy Fuels.

[18] Wei Q., DeBlock R.H., Butts D.M., Choi C., Dunn B. (2020). Pseudocapacitive vanadium-based materials toward high-rate sodium-ion storage. Energy Environ. Mater..

[19] Peng M., Li B., Yan H., Zhang D., Wang X., Xia D., Guo G. (2015). Ruthenium-oxide-coated sodium vanadium fluorophosphate nanowires as high-power cathode materials for sodium-ion batteries. Angew. Chem. Int. Ed..

[20] Wu M., Ni W., Hu J., Ma J. (2019). NASICON-structured NaTi_2_(PO_4_)_3_ for sustainable energy storage. Nano-Micro Lett..

[21] Wei F., Zhang Q., Zhang P., Tian W., Dai K., Zhang L., Mao J., Shao G. (2021). Review—Research progress on layered transition metal oxide cathode materials for sodium ion batteries. J. Electrochem. Soc..

[22] Wang Q., Chu S., Guo S. (2020). Progress on multiphase layered transition metal oxide cathodes of sodium ion batteries. Chin. Chem. Lett..

[23] Guo S., Sun Y., Yi J., Zhu K., Liu P., Zhu Y., Zhu G.-Z., Chen M., Ishida M., Zhou H. (2016). Understanding sodium-ion diffusion in layered P2 and P3 oxides via experiments and first-principles calculations: A bridge between crystal structure and electrochemical performance. NPG Asia Mater..

[24] Wang X., Xiao Z., Han K., Zhang X., Liu Z., Yang C., Meng J., Li M., Huang M., Wei X. (2023). Advances in fine structure optimizations of layered transition-metal oxide cathodes for potassium-ion batteries. Adv. Energy Mater..

[25] Zhu L., Wang H., Sun D., Tang Y., Wang H. (2020). A comprehensive review on the fabrication, modification and applications of Na_3_V_2_(PO_4_)_2_F_3_ cathodes. J. Mater. Chem. A.

[26] Wu J., Xu Y., Sun X., Wang C., Zhang B., Zhao J. (2018). The multiple effects of potassium doping on LiVPO_4_F/C composite cathode material for lithium ion batteries. J. Power Sources.

[27] Xu C., Zhao J., Wang E., Liu X., Shen X., Rong X., Zheng Q., Ren G., Zhang N., Liu X. (2021). A Novel NASICON-typed Na_4_VMn_0.5_Fe_0.5_(PO_4_)_3_ cathode for high-performance na-ion batteries. Adv. Energy Mater..

[28] Xu C., Xiao R., Zhao J., Ding F., Yang Y., Rong X., Guo X., Yang C., Liu H., Zhong B. (2022). Mn-rich phosphate cathodes for Na-ion batteries with superior rate performance. ACS Energy Lett..

[29] Zhou H., Cao Z., Zhou Y., Li J., Ling Z., Fang G., Liang S., Cao X. (2023). Unlocking rapid and robust sodium storage of fluorophosphate cathode via multivalent anion substitution. Nano Energy.

[30] Li P., Gao M., Wang D., Li Z., Liu Y., Liu X., Li H., Sun Y., Liu Y., Niu X. (2023). Optimizing vanadium redox reaction in Na_3_V_2_(PO_4_)_3_ cathodes for sodium-ion batteries by the synergistic effect of additional electrons from heteroatoms. ACS Appl. Mater. Interfaces.

[31] Esparcia E., Joo J., Lee J. (2021). Vanadium oxide bronzes as cathode active materials for non-lithium-based batteries. CrystEngComm.

[32] Heo J.W., Bu H., Hyoung J., Hong S.-T. (2020). Ammonium vanadium bronze, (NH_4_)_2_V_7_O_16_, as a new lithium intercalation host material. Inorg. Chem..

[33] Dong Y., Xu J., Chen M., Guo Y., Zhou G., Li N., Zhou S., Wong C.-P. (2020). Self-assembled NaV_6_O_15_ flower-like microstructures for high-capacity and long-life sodium-ion battery cathode. Nano Energy.

[34] Assadi M.H.N., Okubo M., Yamada A., Tateyama Y. (2020). Possible high-potential ilmenite type Na_1_*M*O_3_ (*M* = V–Ni) cathodes realized by dominant oxygen redox reaction. Phys. Rev. Mater..

[35] Kuang Q., Zhao Y., Dong Y., Fan Q., Lin X., Liu X. (2016). A comparative study of Li_8_NaV_3_(P_2_O_7_)_3_(PO_4_)_2_ and Li_9_V_3_(P_2_O_7_)_3_(PO_4_)_2_: Synthesis, structure and electrochemical properties. J. Power Sources.

[36] Jin T., Liu Y., Li Y., Cao K., Wang X., Jiao L. (2017). Electrospun NaVPO_4_F/C nanofibers as self-standing cathode material for ultralong cycle life Na-ion batteries. Adv. Energy Mater..

[37] Duan W., Zhu Z., Li H., Hu Z., Zhang K., Cheng F., Chen J. (2014). Na_3_V_2_(PO_4_)_3_@C core–shell nanocomposites for rechargeable sodium-ion batteries. J. Mater. Chem. A.

[38] Ren W., Zheng Z., Xu C., Niu C., Wei Q., An Q., Zhao K., Yan M., Qin M., Mai L. (2016). Self-sacrificed synthesis of three-dimensional Na_3_V_2_(PO_4_)_3_ nanofiber network for high-rate sodium–ion full batteries. Nano Energy.

[39] Ni W. (2024). Green, sustainable and massive synthesis of sodium vanadate nanowires toward industrialization. Prog. Progress. Nat. Sci. Mater. Int..

[40] Pan A., Choi D., Zhang J.-G., Liang S., Cao G., Nie Z., Arey B.W., Liu J. (2011). High-rate cathodes based on Li3V2(PO4)3 nanobelts prepared via surfactant-assisted fabrication. J. Power Sources.

[41] Yang Y., Xu S., Zhu W., Xu C., Rui X. (2023). Electrospinning polyanionic materials for high-Rate Na storage. Batter. Supercaps.

[42] Subramanian Y., Oh W., Choi W., Lee H., Jeong M., Thangavel R., Yoon W.-S. (2021). Optimizing high voltage Na_3_V_2_(PO_4_)_2_F_3_ cathode for achieving high rate sodium-ion batteries with long cycle life. Chem. Eng. J..

[43] Shen X., Zhou Q., Han M., Qi X., Li B., Zhang Q., Zhao J., Yang C., Liu H., Hu Y.-S. (2021). Rapid mechanochemical synthesis of polyanionic cathode with improved electrochemical performance for Na-ion batteries. Nat. Commun..

[44] Wang J., Wang Z., Ni J., Li L. (2022). Electrospinning for flexible sodium-ion batteries. Energy Storage Mater..

[45] Rui X., Sun W., Wu C., Yu Y., Yan Q. (2015). An advanced sodium-ion battery composed of carbon coated Na_3_V_2_(PO_4_)_3_ in a porous graphene network. Adv. Mater..

[46] Fang Y., Xiao L., Ai X., Cao Y., Yang H. (2015). Hierarchical carbon framework wrapped Na_3_V_2_(PO_4_)_3_ as a superior high-rate and extended lifespan cathode for sodium-ion batteries. Adv. Mater..

[47] Tertov I.V., Drozhzhin O.A., Alekseeva A.M., Kirsanova M.A., Mironov A.V., Abakumov A.M., Antipov E.V. (2021). β-LiVP_2_O_7_ as a positive electrode material for Li-ion batteries. Electrochim. Acta.

[48] Drozhzhin O.A., Tertov I.V., Alekseeva A.M., Aksyonov D.A., Stevenson K.J., Abakumov A.M., Antipov E.V. (2019). β-NaVP_2_O_7_ as a superior electrode material for Na-ion batteries. Chem. Mater..

[49] Lin X., Zhao Y., Yan D., Han W., Kuang Q., Wen M. (2016). Layered Li-rich vanadium phosphate Li_9_V_3_(P_2_O_7_)_3_(PO_4_)_2_: Cathode and anode materials for lithium-ion batteries. Electrochim. Acta.

[50] Saravanan K., Mason C.W., Rudola A., Wong K.H., Balaya P. (2013). The first report on excellent cycling stability and superior rate capability of Na_3_V_2_(PO_4_)_3_ for sodium ion batteries. Adv. Energy Mater..

[51] Min X., Huo H., Li R., Zhou J., Hu Y., Dai C. (2016). Cycling stability of Li_3_V_2_(PO_4_)_3_/C cathode in a broad electrochemical window. J. Electroanal. Chem..

[52] Jia Y., Wu Y., Li L., Song L., Gao J. (2023). Monoclinic Na_2_VOP_2_O_7_: A 4V-class cost-effective cathode for sodium-ion batteries. Mater. Today Phys..

[53] Kim J., Park I., Kim H., Park K.-Y., Park Y.-U., Kang K. (2016). Tailoring a new 4V-class cathode material for Na-ion batteries. Adv. Energy Mater..

[54] Deng C., Zhang S., Zhao B. (2016). First exploration of ultrafine Na_7_V_3_(P_2_O_7_)_4_ as a high-potential cathode material for sodium-ion battery. Energy Storage Mater..

[55] Lim S.Y., Kim H., Chung J., Lee J.H., Kim B.G., Choi J.-J., Chung K.Y., Cho W., Kim S.-J., Goddard W.A. (2014). Role of intermediate phase for stable cycling of Na_7_V_4_(P_2_O_7_)_4_PO_4_ in sodium ion battery. Proc. Natl. Acad. Sci. USA.

[56] Deng C., Zhang S. (2014). 1D Nanostructured Na_7_V_4_(P_2_O_7_)_4_(PO_4_) as high-potential and superior-performance cathode material for sodium-ion batteries. ACS Appl. Mater. Interfaces.

[57] Park Y.-U., Seo D.-H., Kim H., Kim J., Lee S., Kim B., Kang K. (2014). A family of high-performance cathode materials for Na-ion batteries, Na_3_(VO_1−*x*_PO_4_)_2_F_1+2*x*_ (0 ≤ x ≤ 1): Combined first-principles and experimental study. Adv. Funct. Mater..

[58] Barker J., Saidi M.Y., Swoyer J.L. (2003). Electrochemical insertion properties of the novel lithium vanadium fluorophosphate, LiVPO_4_ F. J. Electrochem. Soc..

[59] Park Y.-U., Seo D.-H., Kwon H.-S., Kim B., Kim J., Kim H., Kim I., Yoo H.-I., Kang K. (2013). A new high-energy cathode for a Na-ion battery with ultrahigh stability. J. Am. Chem. Soc..

[60] Zhang X., Zhang Z., Xu S., Xu C., Rui X. (2023). Advanced vanadium oxides for sodium-ion batteries. Adv. Funct. Mater..

[61] Niu X., Li N., Chen Y., Zhang J., Yang Y., Tan L., Wu J., Guo L., Zhu Y. (2023). Structure evolution of V_2_O_5_ as electrode materials for metal-ion batteries. Batter. Supercaps.

[62] Yao J., Li Y., Massé R.C., Uchaker E., Cao G. (2018). Revitalized interest in vanadium pentoxide as cathode material for lithium-ion batteries and beyond. Energy Storage Mater..

[63] Liu Q., Li Z.-F., Liu Y., Zhang H., Ren Y., Sun C.-J., Lu W., Zhou Y., Stanciu L., Stach E.A. (2015). Graphene-modified nanostructured vanadium pentoxide hybrids with extraordinary electrochemical performance for Li-ion batteries. Nat. Commun..

[64] Zhu L., Ge P., Xie L., Miao Y., Cao X. (2020). Doped-Li_1+*x*_V_3_O_8_ as cathode materials for lithium-ion batteries: A mini review. Electrochem. Commun..

[65] Esparcia E.A., Chae M.S., Ocon J.D., Hong S.-T. (2018). Ammonium vanadium bronze (NH_4_V_4_O_10_) as a high-capacity cathode material for nonaqueous magnesium-ion batteries. Chem. Mater..

[66] Christensen C.K., Sørensen D.R., Hvam J., Ravnsbæk D.B. (2019). Structural evolution of disordered Li*_x_*V_2_O_5_ bronzes in V_2_O_5_ cathodes for Li-ion batteries. Chem. Mater..

[67] Yang D., Zhang D., Wu H., Pei C., Xiao T., Ma H., Ni S. (2023). Construction of a LiVO_3_/C core–shell structure for high-rate lithium storage. New J. Chem..

[68] Fu X., Pu X., Wang H., Zhao D., Liu G., Zhao D., Chen Z. (2019). Understanding capacity fading of the LiVO_3_ cathode material by limiting the cutoff voltage. Phys. Chem. Chem. Phys..

[69] Zheng H., Yang Y., Liu X., Guo Z., Feng C. (2017). Controllable synthesis of FeVO_4_@TiO_2_ nanostructures as anode for lithium ion battery. J. Nanopart. Res..

[70] Zheng H., Zhang Q., Gao H., Sun W., Zhao H., Feng C., Mao J., Guo Z. (2019). Synthesis of porous MoV_2_O_8_ nanosheets as anode material for superior lithium storage. Energy Storage Mater..

[71] Zhang C., Song H., Liu C., Liu Y., Zhang C., Nan X., Cao G. (2015). Fast and reversible Li ion insertion in carbon-encapsulated Li_3_VO_4_ as anode for lithium-ion battery. Adv. Funct. Mater..

[72] Liao C., Zhang Q., Zhai T., Li H., Zhou H. (2017). Development and perspective of the insertion anode Li_3_VO_4_ for lithium-ion batteries. Energy Storage Mater..

[73] Shi H.-Y., Jia Z., Wu W., Zhang X., Liu X.-X., Sun X. (2020). The development of vanadyl phosphate cathode materials for energy storage systems: A review. Chem. Eur. J..

[74] Hu P., Wang X., Ma J., Zhang Z., He J., Wang X., Shi S., Cui G., Chen L. (2016). NaV_3_(PO_4_)_3_/C nanocomposite as novel anode material for Na-ion batteries with high stability. Nano Energy.

[75] Miao X., Li C., Chu W., Wu P., Tong D.G. (2015). Li_9_V_3_(P_2_O_7_)_3_(PO_4_)_2_ nanotubes fabricated by a simple molten salt approach with excellent cycling stability and enhanced rate capability in lithium-ion batteries. RSC Adv..

[76] Lv Z., Ling M., Yue M., Li X., Song M., Zheng Q., Zhang H. (2021). Vanadium-based polyanionic compounds as cathode materials for sodium-ion batteries: Toward high-energy and high-power applications. J. Energy Chem..

[77] Li Y., Liang X., Zhong G., Wang C., Wu S., Xu K., Yang C. (2020). Fiber-shape Na_3_V_2_(PO_4_)_2_F_3_@N-doped carbon as a cathode material with enhanced cycling stability for Na-ion batteries. ACS Appl. Mater. Interfaces.

[78] Liu Q., Wang D., Yang X., Chen N., Wang C., Bie X., Wei Y., Chen G., Du F. (2015). Carbon-coated Na_3_V_2_(PO_4_)_2_F_3_ nanoparticles embedded in a mesoporous carbon matrix as a potential cathode material for sodium-ion batteries with superior rate capability and long-term cycle life. J. Mater. Chem. A.

[79] Ruan Y.-L., Wang K., Song S.-D., Han X., Cheng B.-W. (2015). Graphene modified sodium vanadium fluorophosphate as a high voltage cathode material for sodium ion batteries. Electrochim. Acta.

[80] Lin X., Huang J., Tan H., Huang J., Zhang B. (2019). K_3_V_2_(PO_4_)_2_F_3_ as a robust cathode for potassium-ion batteries. Energy Storage Mater..

[81] Beltrop K., Beuker S., Heckmann A., Winter M., Placke T. (2017). Alternative electrochemical energy storage: Potassium-based dual-graphite batteries. Energy Environ. Sci..

[82] Xu Y., Titirici M., Chen J., Cora F., Cullen P.L., Edge J.S., Fan K., Fan L., Feng J., Hosaka T. (2023). 2023 roadmap for potassium-ion batteries. J. Phys. Energy.

[83] Wang Y.-Y., Hou B.-H., Guo J.-Z., Ning Q.-L., Pang W.-L., Wang J., Lü C.-L., Wu X.-L. (2018). An ultralong lifespan and low-temperature workable sodium-ion full battery for stationary energy storage. Adv. Energy Mater..

[84] Krishna M.M., Jayasree G., Mallu R.R., Basak P. (2023). Impact of electrolyte composition on the performance of Na_3_(VOPO_4_)_2_F Cathodes: A Comprehensive Case Study. ACS Appl. Energy Mater..

[85] Liu T., Zhang Y., Chen C., Lin Z., Zhang S., Lu J. (2019). Sustainability-inspired cell design for a fully recyclable sodium ion battery. Nat. Commun..

[86] Samarin A.S., Ivanov A.V., Fedotov S.S. (2023). Toward efficient recycling of vanadium phosphate-based sodium-ion batteries: A review. Clean Technol..

[87] Yue Y., Liang H. (2017). Micro- and nano-structured vanadium pentoxide (V_2_O_5_) for electrodes of lithium-ion batteries. Adv. Energy Mater..

[88] Rui X., Lu Z., Yu H., Yang D., Hng H.H., Lim T.M., Yan Q. (2013). Ultrathin V_2_O_5_ nanosheet cathodes: Realizing ultrafast reversible lithium storage. Nanoscale.

[89] Ding Y.-L., Wen Y., Wu C., van Aken P.A., Maier J., Yu Y. (2015). 3D V_6_O_13_ nanotextiles assembled from interconnected nanogrooves as cathode materials for high-energy lithium ion batteries. Nano Lett..

[90] Ren M.M., Zhou Z., Gao X.P., Peng W.X., Wei J.P. (2008). Core−shell Li_3_V_2_(PO_4_)_3_@C composites as cathode materials for lithium-ion batteries. J. Phys. Chem. C.

[91] Ni Q., Zheng L., Bai Y., Liu T., Ren H., Xu H., Wu C., Lu J. (2020). An extremely fast charging Li_3_V_2_(PO_4_)_3_ cathode at a 4.8 V cutoff voltage for Li-ion batteries. ACS Energy Lett..

[92] Kuang Q., Xu J., Zhao Y., Chen X., Chen L. (2011). Layered monodiphosphate Li_9_V_3_(P_2_O_7_)_3_(PO_4_)_2_: A novel cathode material for lithium-ion batteries. Electrochim. Acta.

[93] Dugas R., Zhang B., Rozier P., Tarascon J.M. (2016). Optimization of Na-ion battery systems based on polyanionic or layered positive electrodes and carbon anodes. J. Electrochem. Soc..

[94] Emery N., Baddour-Hadjean R., Batyrbekuly D., Laïk B., Bakenov Z., Pereira-Ramos J.-P. (2018). γ-Na_0.96_V_2_O_5_: A new competitive cathode material for sodium-ion batteries synthesized by a soft chemistry route. Chem. Mater..

[95] Pan Z.-T., He Z.-H., Hou J.-F., Kong L.-B. (2022). Sodium vanadium hexacyanoferrate as a high-rate capability and long-life cathode material for Na-ion batteries. J. Energy Storage.

[96] Baster D., Kondracki Ł., Oveisi E., Trabesinger S., Girault H.H. (2021). Prussian blue analogue—Sodium–vanadium hexacyanoferrate as a cathode material for Na-ion batteries. ACS Appl. Energy Mater..

[97] Yin X., Sarkar S., Shi S., Huang Q.-A., Zhao H., Yan L., Zhao Y., Zhang J. (2020). Recent progress in advanced organic electrode materials for sodium-ion batteries: Synthesis, mechanisms, challenges and perspectives. Adv. Funct. Mater..

[98] Cao X., Zhou J., Pan A., Liang S. (2020). Recent advances in phosphate cathode materials for sodium-ion batteries. Acta Phys.-Chim. Sin..

[99] Choi J.W., Aurbach D. (2016). Promise and reality of post-lithium-ion batteries with high energy densities. Nat. Rev. Mater..

[100] Tepavcevic S., Xiong H., Stamenkovic V.R., Zuo X., Balasubramanian M., Prakapenka V.B., Johnson C.S., Rajh T. (2012). Nanostructured bilayered vanadium oxide electrodes for rechargeable sodium-ion batteries. ACS Nano.

[101] Kovrugin V.M., David R., Chotard J.-N., Recham N., Masquelier C. (2018). A High voltage cathode material for sodium batteries: Na_3_V(PO_4_)_2_. Inorg. Chem..

[102] Fang R., Olchowka J., Pablos C., Camacho P.S., Carlier D., Croguennec L., Cassaignon S. (2022). Effect of the particles morphology on the electrochemical performance of Na_3_V_2_(PO_4_)_2_F_3-y_O_y_. Batter. Supercaps.

[103] Fang R., Olchowka J., Pablos C., Bianchini Nuernberg R., Croguennec L., Cassaignon S. (2022). Impact of the F^–^ for O^2–^ Substitution in Na_3_V_2_(PO_4_)_2_F_3–*y*_O*_y_* on their transport properties and electrochemical performance. ACS Appl. Energy Mater..

[104] Yang Z., Li G., Sun J., Xie L., Jiang Y., Huang Y., Chen S. (2020). High performance cathode material based on Na_3_V_2_(PO_4_)_2_F_3_ and Na_3_V_2_(PO_4_)_3_ for sodium-ion batteries. Energy Storage Mater..

[105] Fang Y., Liu Q., Xiao L., Rong Y., Liu Y., Chen Z., Ai X., Cao Y., Yang H., Xie J. (2018). A fully sodiated NaVOPO_4_ with layered structure for high-voltage and long-lifespan sodium-ion batteries. Chem.

[106] Qi Y., Mu L., Zhao J., Hu Y.-S., Liu H., Dai S. (2015). Superior Na-storage performance of low-temperature-synthesized Na_3_(VO_1−*x*_PO_4_)_2_F_1+2*x*_ (0 ≤ *x* ≤ 1) nanoparticles for Na-ion batteries. Angew. Chem. Int. Ed..

[107] Shraer S.D., Luchinin N.D., Trussov I.A., Aksyonov D.A., Morozov A.V., Ryazantsev S.V., Iarchuk A.R., Morozova P.A., Nikitina V.A., Stevenson K.J. (2022). Development of vanadium-based polyanion positive electrode active materials for high-voltage sodium-based batteries. Nat. Commun..

[108] Jin H., Dong J., Uchaker E., Zhang Q., Zhou X., Hou S., Li J., Cao G. (2015). Three dimensional architecture of carbon wrapped multilayer Na_3_V_2_O_2_(PO_4_)_2_F nanocubes embedded in graphene for improved sodium ion batteries. J. Mater. Chem. A.

[109] Cai Y., Cao X., Luo Z., Fang G., Liu F., Zhou J., Pan A., Liang S. (2018). Caging Na_3_V_2_(PO_4_)_2_F_3_ microcubes in cross-linked graphene enabling ultrafast sodium storage and long-term cycling. Adv. Sci..

[110] He Y., Li H. (2023). Recent research process of carbon engineering on Na_3_V_2_(PO_4_)_3_ for sodium-ion battery cathodes: A mini review. Electron. Mater..

[111] Liu J., Tang K., Song K., van Aken P.A., Yu Y., Maier J. (2014). Electrospun Na_3_V_2_(PO_4_)_3_/C nanofibers as stable cathode materials for sodium-ion batteries. Nanoscale.

[112] Wu L., Hao Y., Shi S., Zhang X., Li H., Sui Y., Yang L., Zhong S. (2018). Controllable synthesis of Na_3_V_2_(PO_4_)_3_/C nanofibers as cathode material for sodium-ion batteries by electrostatic spinning. Front. Chem..

[113] Liang L., Li X., Zhao F., Zhang J., Liu Y., Hou L., Yuan C. (2021). Construction and operating mechanism of high-rate Mo-doped Na_3_V_2_(PO_4_)_3_@C nanowires toward practicable wide-temperature-tolerance Na-ion and hybrid Li/Na-ion batteries. Adv. Energy Mater..

[114] Luo L., Cheng B., Chen Y., Chen S., Liu G., Zhuo H. (2020). Electrospun Na_3_V_2_(PO_4_)_3_/C nanofibers as self-standing cathode material for high performance sodium ion batteries. Mater. Res. Express.

[115] Xu D., Chen R., Chen B., Zhou S., Zhang Y., Chang Z., Pan A. (2023). High-performance flexible sodium-ion batteries enabled by high-voltage sodium vanadium fluorophosphate nanorod arrays. Sci. China Mater..

[116] Wang D., Wei Q., Sheng J., Hu P., Yan M., Sun R., Xu X., An Q., Mai L. (2016). Flexible additive free H_2_V_3_O_8_ nanowire membrane as cathode for sodium ion batteries. Phys. Chem. Chem. Phys..

[117] Clites M., Hart J.L., Taheri M.L., Pomerantseva E. (2020). Annealing-assisted enhancement of electrochemical stability of Na-preintercalated bilayered vanadium oxide electrodes in Na-ion batteries. ACS Appl. Energy Mater..

[118] Min X., Xiao J., Fang M., Wang W., Zhao Y., Liu Y., Abdelkader A.M., Xi K., Kumar R.V., Huang Z. (2021). Potassium-ion batteries: Outlook on present and future technologies. Energy Environ. Sci..

[119] Zhang K.-Y., Gu Z.-Y., Ang E.H., Guo J.-Z., Wang X.-T., Wang Y., Wu X.-L. (2022). Advanced polyanionic electrode materials for potassium-ion batteries: Progresses, challenges and application prospects. Mater. Today.

[120] Kim H., Seo D.-H., Bianchini M., Clément R.J., Kim H., Kim J.C., Tian Y., Shi T., Yoon W.-S., Ceder G. (2018). A new strategy for high-voltage cathodes for K-ion batteries: Stoichiometric KVPO_4_F. Adv. Energy Mater..

[121] Fedotov S.S., Samarin A.S., Antipov E.V. (2020). KTiOPO_4_-structured electrode materials for metal-ion batteries: A review. J. Power Sources.

[122] Deng L., Niu X., Ma G., Yang Z., Zeng L., Zhu Y., Guo L. (2018). Layered potassium vanadate K_0.5_V_2_O_5_ as a cathode material for nonaqueous potassium ion batteries. Adv. Funct. Mater..

[123] Zhang Y., Niu X., Tan L., Deng L., Jin S., Zeng L., Xu H., Zhu Y. (2020). K_0.83_V_2_O_5_: A new layered compound as a stable cathode material for potassium-ion batteries. ACS Appl. Mater. Interfaces.

[124] Niu X., Qu J., Hong Y., Deng L., Wang R., Feng M., Wang J., Zeng L., Zhang Q., Guo L. (2021). High-performance layered potassium vanadium oxide for K-ion batteries enabled by reduced long-range structural order. J. Mater. Chem. A.

[125] Clites M., Hart J.L., Taheri M.L., Pomerantseva E. (2018). Chemically preintercalated bilayered K*_x_*V_2_O_5_·*n*H_2_O nanobelts as a high-performing cathode material for K-Ion batteries. ACS Energy Lett..

[126] Zhu Y.-R., Cao K., Chen F., Dong J.-M., Ren N.-Q., Chen C.-H. (2023). Fine valence regulation of hydrated vanadium oxide as a novel cathode for stable potassium-ion storage. Chem. Commun..

[127] Zhu Y.-H., Zhang Q., Yang X., Zhao E.-Y., Sun T., Zhang X.-B., Wang S., Yu X.-Q., Yan J.-M., Jiang Q. (2019). Reconstructed orthorhombic V_2_O_5_ polyhedra for fast ion diffusion in K-Ion batteries. Chem.

[128] Han J., Li G.-N., Liu F., Wang M., Zhang Y., Hu L., Dai C., Xu M. (2017). Investigation of K_3_V_2_(PO_4_)_3_/C nanocomposites as high-potential cathode materials for potassium-ion batteries. Chem. Commun..

[129] Xie C., Liu X., Han J., Lv L., Zhou X., Han C., You Y. (2022). Pomegranate-like KVPO_4_F@C microspheres as high-volumetric-energy-density cathode for potassium-ion batteries. Small.

[130] Liao J., Zhang X., Zhang Q., Hu Q., Li Y., Du Y., Xu J., Gu L., Zhou X. (2022). Synthesis of KVPO_4_F/carbon porous single crystalline nanoplates for high-rate potassium-ion batteries. Nano Lett..

[131] Gao Y., Li W., Ou B., Zhang S., Wang H., Hu J., Kang F., Zhai D. (2023). A dilute fluorinated phosphate electrolyte enables 4.9 V-class potassium ion full batteries. Adv. Funct. Mater..

[132] Liao J., Hu Q., Che B., Ding X., Chen F., Chen C. (2019). Competing with other polyanionic cathode materials for potassium-ion batteries via fine structure design: New layered KVOPO_4_ with a tailored particle morphology. J. Mater. Chem. A.

[133] Liao J., Hu Q., Mu J., He X., Wang S., Chen C. (2019). A vanadium-based metal–organic phosphate framework material K_2_[(VO)_2_(HPO_4_)_2_(C_2_O_4_)] as a cathode for potassium-ion batteries. Chem. Commun..

[134] Hameed A.S., Katogi A., Kubota K., Komaba S. (2019). A layered inorganic–organic open framework material as a 4 V positive electrode with high-rate performance for K-ion batteries. Adv. Energy Mater..

[135] He X.-D., Liao J.-Y., Chen T., Zhang L.-M., Ding X., Wang J.-R., Wang S., Wen Z.-Y., Chen C.-H. (2021). Spray drying derived wrinkled pea-shaped carbon-matrixed KVP_2_O_7_ as a cathode material for potassium-ion batteries. J. Alloys Compd..

